# Alzheimer’s Disease, Obesity, and Type 2 Diabetes: Focus on Common Neuroglial Dysfunctions (Critical Review and New Data on Human Brain and Models)

**DOI:** 10.3390/brainsci14111101

**Published:** 2024-10-30

**Authors:** Adolfo Toledano, Arantxa Rodríguez-Casado, María Isabel Älvarez, Adolfo Toledano-Díaz

**Affiliations:** 1 Instituto Cajal, CSIC, 28002 Madrid, Spain; arantxa.rodriguezcasado@gmail.com (A.R.-C.); miavmasueco@hotmail.com (M.I.Ä.); 2 Departamento Reproducción Animal, INIA-CSIC, 28040 Madrid, Spain; toledano@inia.csic.es

**Keywords:** Alzheimer’s disease (AD), obesity, type 2 diabetes (T2D), pathogenic mechanisms, neuroglia, astroglia, oligodendroglia, microglia, neuroinflammation, neuroglial dysfunctions, neuropathology, neuroglial therapy

## Abstract

Background/Objectives. Obesity, type 2 diabetes (T2D), and Alzheimer’s disease (AD) are pathologies that affect millions of people worldwide. They have no effective therapy and are difficult to prevent and control when they develop. It has been known for many years that these diseases have many pathogenic aspects in common. We highlight in this review that neuroglial cells (astroglia, oligodendroglia, and microglia) play a vital role in the origin, clinical–pathological development, and course of brain neurodegeneration. Moreover, we include the new results of a T2D-AD mouse model (APP+PS1 mice on a high-calorie diet) that we are investigating. Methods. Critical bibliographic revision and biochemical neuropathological study of neuroglia in a T2D-AD model. Results. T2D and AD are not only “connected” by producing complex pathologies in the same individual (obesity, T2D, and AD), but they also have many common pathogenic mechanisms. These include insulin resistance, hyperinsulinemia, hyperglycemia, oxidative stress, mitochondrial dysfunction, and inflammation (both peripheral and central—or neuroinflammation). Cognitive impairment and AD are the maximum exponents of brain neurodegeneration in these pathological processes. both due to the dysfunctions induced by metabolic changes in peripheral tissues and inadequate neurotoxic responses to changes in the brain. In this review, we first analyze the common pathogenic mechanisms of obesity, T2D, and AD (and/or cerebral vascular dementia) that induce transcendental changes and responses in neuroglia. The relationships between T2D and AD discussed mainly focus on neuroglial responses. Next, we present neuroglial changes within their neuropathological context in diverse scenarios: (a) aging involution and neurodegenerative disorders, (b) human obesity and diabetes and obesity/diabetes models, (c) human AD and in AD models, and (d) human AD-T2D and AD-T2D models. An important part of the data presented comes from our own studies on humans and experimental models over the past few years. In the T2D-AD section, we included the results of a T2D-AD mouse model (APP+PS1 mice on a high-calorie diet) that we investigated, which showed that neuroglial dysfunctions (astrocytosis and microgliosis) manifest before the appearance of amyloid neuropathology, and that the amyloid pathology is greater than that presented by mice fed a normal, non-high-caloric diet A broad review is finally included on pharmacological, cellular, genic, and non-pharmacological (especially diet and lifestyle) neuroglial-related treatments, as well as clinical trials in a comparative way between T2D and AD. These neuroglial treatments need to be included in the multimodal/integral treatments of T2D and AD to achieve greater therapeutic efficacy in many millions of patients. Conclusions. Neuroglial alterations (especially in astroglia and microglia, cornerstones of neuroinflammation) are markedly defining brain neurodegeneration in T2D and A, although there are some not significant differences between each of the studied pathologies. Neuroglial therapies are a very important and p. promising tool that are being developed to prevent and/or treat brain dysfunction in T2D-AD. The need for further research in two very different directions is evident: (a) characterization of the phenotypic changes of astrocytes and microglial cells in each region of the brain and in each phase of development of each isolated and associated pathology (single-cell studies are mandatory) to better understand the pathologies and define new therapeutic targets; (b) studying new therapeutic avenues to normalize the function of neuroglial cells (preventing neurotoxic responses and/or reversing them) in these pathologies, as well as the phenotypic characteristics in each moment of the course and place of the neurodegenerative process.

## 1. Introduction

Alzheimer’s disease (AD) affects, and will affect, an exceptionally large number of people in the world. People with dementia will increase from 57 million cases globally in 2019 to 152 (130.8 to 175.9) million cases in 2050, according to a study by the Global Burden of Disease Organization in 2019 [1] AD is a syndrome, that is, with various etiologies, that can be considered as a dual disorder: clinically, it courses with dementia (disorder presenting a constellation of signs and symptoms of cognitive decline, including impaired memory/ies, faulty executive functions and judgment, and behavioral alterations) (see DMS V [2,3,4,5], and pathologically, it progresses with neurodegeneration (displaying degenerative neuronal changes, reactive changes in neuroglial cells, neuronal network alterations, and the accumulation of misfolded protein deposits (intraneuronal neurofibrillary tangles and extraneuronal amyloid deposits) [6,7,8,9,10,11,12]. These pathological disturbances are accompanied by oxidative stress, neuroinflammatory processes, neurotransmission alterations, neuronal–neuroglial communication dysfunctions, synaptic involution, and neuronal and neuroglial metabolic changes [13,14,15,16,17,18,19,20,21,22,23,24,25].

Important clinical studies have shown that AD is associated with other systemic pathologies, especially those caused by carbohydrate and lipid metabolic disorders considered risk factors for AD [26,27,28,29,30,31,32]. Likewise, pathological studies have shown neuroinflammatory alterations similar to those observed in AD [26]. Obesity and type 2 diabetes (T2D) can lead to cognitive decline and neurodegeneration [32,33]. Insulin resistance is a key process in both these metabolic processes [32,33,34,35,36,37].

Neurons and neuronal circuits are the ultimate targets of neurodegenerative processes that lead to brain neuropathological deterioration and clinical dementia. These neuronal deleterious effects are the main basis of clinical manifestations in these neurodegenerative processes. However, with a variable importance, over the last century and a half, the idea that neuroglial cells, constant companions of the life and functions of neurons as well as their adaptive capacities to carry out the coordination of internal responses (of the organs and systems) as external of a being, are absolutely fundamental to both the maintenance of health and the development of diseases (cerebral and systemic) [19,20]. Neuroglial cell alterations are of paramount importance in brain deterioration. Astrocytes, oligodendrocytes, and microglial cells play a stellar role in the genesis and development of brain neurodegeneration in systemic metabolic diseases and neurodegenerative diseases such as AD [7,19,20,31,32,38,39,40,41,42,43,44].

This review will deal with the concurrent neuroglial alterations that appear in the brain of both AD and systemic metabolic diseases related to glucose dysmetabolism (obesity and type 2 diabetes) in the context of a common way to arrive at a profound cognitive decline late in life and a deep/aberrant neuropathological involution. The common mechanisms in the genesis and course of the AD and T2D neuropathological processes are presented (Section 2.1—common accepted mechanisms among these pathologies) but without forgetting the interpretations of research results that do not consider the existence of a closed pathogenic interrelationship (Section 2.2—controversies on pathological AD, obesity, and T2D relationships). In these initial chapters, Section 2.1 and Section 2.2, we wanted to present the neuropathological scenarios in which the important neuroglial alterations develop and are the main basis of the pathogenies of these studied diseases. A summary of glial–neuron interrelationships in healthy young brains is presented in Section 3.1, in addition to the reactive neuroglial changes in aging and diseases (Section 3.2): general reactive neuroglial states (Section 3.2.1) and the specific presentations in aging (Section 3.2.1), as well as in reviewed pathological disorders, AD (Section 3.2.2) and T2D (Section 3.2.3). A wide review of the therapeutical possibilities acting on neuroglial abnormal responses is then analyzed (Section 4). A discussion on the controversies in the field, divergent pathogenic mechanisms, and new lines of research will also be pointed out (Section 5) to arrive at an effective preventive and curative treatment against pathological synergies in this complex of concomitant disorders of cognitive impairment (AD/obesity/type 2 diabetes/metabolic dysfunctions).

## 2. Common Pathological Mechanisms in AD, Obesity, and Type 2 Diabetes

### 2.1. Common Accepted Pathogenic Mechanisms Among These Pathologies

Alzheimer’s disease and type 2 diabetes mellitus are two diseases with a high incidence today. They share similar key pathophysiological mechanisms, suggesting a potential causal relationship or intricate linking between them [32]. In fact, Alzheimer-associated mellitus diabetes is also known as type 3 diabetes mellitus [45]. An interesting work by Erol, 2008 [46], shows that AD could be considered a metabolic disease of the CNS. The author points out various pathogenic AD mechanisms that can be found in the pathogenic descriptions of T2D and cognitive decline in T2D. Brain glucose utilization is reduced in the early stages of AD, when hyperinsulinemia, a characteristic finding of insulin resistance, as well as glucose levels, is increased. Insufficient insulin signaling impairs the oxidative stress regulation and decreases astrocytic energy substrates and the antioxidant supply of neurons, and glucose excess (associated with hyperleptinemia) may worsen the reduced astrocytic energy supply and the ongoing neuroinflammation via the inhibition of AMP-activated protein kinase (AMPK).

**Figure 1:**
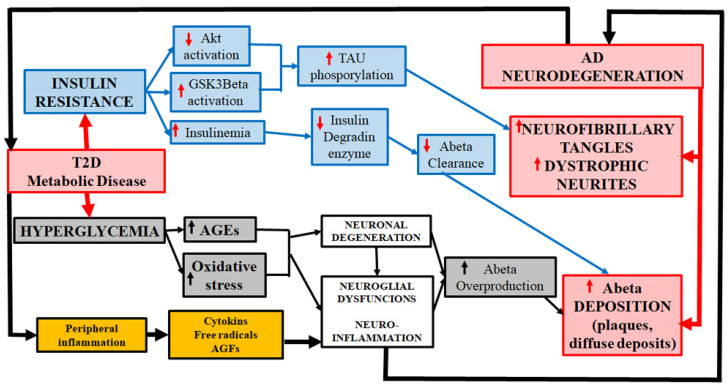
Pathways of neurodegeneration in the diabetic brain. Critical links between type 2 diabetes (T2D) and Alzheimer’s disease (AD). The two main neuropathological manifestations in AD are intracellular deposits of hyperphosphorylated tau protein (phospho-tau), which give rise to dysfunctional dystrophic neurites and neurons bearing neurofibrillary tangles, and extracellular deposits of beta-amyloid protein. Both pathologic alterations are facilitated by insulin resistance, the major cause of T2D. Insulin resistance, through the activation of Akt and GSK3-beta, increases the phosphorylation of the tau protein. Moreover, through increased insulinemia, the clearance of the beta-amyloid protein decreases by deactivating the insulin-degrading enzyme (IDE). On the other hand, hyperglycemia induced by T2D leads to an overproduction of beta-amyloid protein (Abeta) that is deposited, to a substantial extent, as plaques and/or diffuse amyloid. Chronic hyperglycemia generates advanced glycation end products (AGEs) and oxidative stress that induces neuronal degeneration and neuronal dysfunctions/neuroinflammation (that also cause higher neurodegeneration). Related peripheral inflammation induced by T2D, through cytokines, free radicals, and AGEs, increases neuroinflammation, key pathogenic mechanism for AD neurodegeneration. Phospho-tau and beta-amyloid also activate these neurodegenerative processes in a vicious cycle. T2D induces AD, and AD induces T2D and related peripheral inflammation (based on [32,48]).

Obesity is a pathological situation (although “healthy obesity” is still considered to exist in some instances) that precedes the development of T2D [32]. Central to this association of AD-obesity-T2D is insulin resistance, a key factor leading to decreased insulin sensitivity, hyperglycemia, and impaired glucose uptake [32,47,48,49,50,51,52,53,54] (Figure 1). The brains of patients suffering AD or T2D are altered by energy deficits and neuronal/neuroglial damage. These brains show common genetic–epigenetic, biochemical, and physiological characteristics such as inflammation, mitochondrial dysfunction, oxidative stress, and the formation of advanced glycation end products (reviewed in Rodriguez-Casado et al., 2017 [32]). Furthermore, many of the risk factors for suffering from AD or T2D are the same (bad lifestyle habits, incorrect forms of nutrition and diets, a sedentary lifestyle, and a lack of physical exercise). And non-pharmacological approaches to counteract these risk factors are also similar. In the therapeutic aspect, it is relevant to point out that there are many preclinical and clinical/trial studies that indicate that antidiabetic therapies are beneficial in the treatment of AD and cognitive impairment [55,56,57,58].

There are different mechanisms proposed to explain how diabetes contributes to AD: glucose dysregulation, resulting in hyperglycemia and insulin resistance [59,60,61,62] (Figure 1); oxidative stress and peripheral inflammation [63,64,65]; mitochondrial dysfunction [66,67]); dysfunctionality of the blood–brain barrier (BBB), which compromises nutrient and oxygen delivery to the brain and impairs the clearance of Aβ-amyloid [68]; neuronal alterations, inducing amyloid and tau deposition [30,51]; and, the most important for many authors, the firing of the devasting neuroinflammatory processes [69,70,71,72] consecutive to the induced neuroglial dysfunctions. Reactive oxygen and nitrogen species are important molecules to trigger degenerative/involutive changes in cells of peripheral tissues as well as in neurons and neuroglial cells of the CNS [73,74]. Diabetes is associated with peripheral chronic low-grade inflammation, characterized by increased levels of pro-inflammatory cytokines. These toxic molecules enter the brain, provoking reactive responses in glial cells, the origin of brain neuroinflammation that plays a key role in AD development/progression, contributing to neuronal dysfunction and neurodegeneration [65,72,73,74,75,76,77,78,79,80]. All these pathogenic mechanisms seem to be involved to different degrees to end up producing different pathogenic situations that clearly fall into the clinical diagnosis of AD. Oxidative/nitrosative stress is involved in molecular pathways that appear to contribute to immune imbalance and cytokine dysregulation, which is associated with “inflammageing” or parainflammation [81,82,83]. Mitochondrial dysfunctions and amyloid and phospho-tau formation and deposition are clearly consequences of these pathogenic mechanisms. Neuroinflammation is a key mechanism or cornerstone of the theories that explain the inducing role of T2D and related disorders in AD development and/or increasing pathogenic progression [32,39,41,42,44,51].

Apart from the clear implications of obesity and T2D with AD, which indicate a twinning or intertwining of pathogenic mechanisms to end in neurodegeneration/dementia typical of AD, a pathological entity called “diabetes-related dementia” has also been described. This disorder presents a slow progression of cognitive decline and less or no amyloid accumulation [84]. However, insulin resistance, elevated inflammatory cytokines, oxidative stress, and advanced glycation end products are associated with this type of dementia [84]. Although it seems to be dementia different from AD, we could consider it as a stage prior to the development of AD, in the same way that mild cognitive impairment is observed as a pre-AD situation. We cannot forget that AD is a syndrome with quite different etiologies and pathogenic courses, some more or less related to disturbances of carbohydrate and lipid metabolism.

Many of the studies that attempt to clarify the origin of the alterations that lead to metabolic alterations that cause obesity and T2D, and subsequently to cognitive decline/AD, lean toward one of the two main pathogenic mechanisms: (i) genetic individual/familial mandatory development of altered biomolecular mechanisms that give rise to neuropathology or (ii) epigenetic alterations and/or changes induced by an increase in the intensity of risk factors. In this sense, it is work highlighting how the intake of high concentrations of carbohydrates can alter the function of various hypothalamic centers (favored by a special permeability of the BBB), causing altered responses in the regulation of carbohydrate metabolism [85]. The arcuate nucleus of the hypothalamus controls energy expenditure and intake. In this function, their neurons integrate circulating hormonal and metabolic signals. The circulating molecules serving of drivers arrive to arcuate neurons through the vasculature of the median eminence, which lacks a typical blood–brain barrier, and the tanycytes (subtype or astroglia) that actively transport these molecules. In this regulatory system of brain energy metabolism, the special astroglial cells of the median eminence have a very predominant role [86,87,88,89,90,91]. These initial changes in the energy-regulatory function propagate to other regions of the brain in a manner that is not well understood so far, but it has been demonstrated how the persistence of the actions of the risk factors produces metabolic/neuropathological alterations in other regions of the brain. CNS (hippocampus and cerebral cortex). In this induction of neuropathological alterations in other brain regions, neuroinflammation is a main part. Astroglia and microglia activation is always present [92,93,94,95]. Inactivation of the microglia response protects from induced diabetogenic alterations [96]. High glucose impairs the astrocytic response, endothelial cell responses, and normal BBB functions [97].

Among all the above-mentioned pathogenic mechanisms, we want to highlight the role that the responses of neuroglial cells have. We must not forget that when faced with changes in the neuronal environment, glial cells respond in a dual manner. On the one hand, they try to restore the homeostasis of the environment, and on the other hand, they try to eliminate the disturbing agents in the environment as well as the affected cells that are not recoverable or could be toxic to the healthy neuron–neuroglial team of the brain. Taking this duality into account is particularly important when designing treatments to correct neuroglial responses in these pathologies.

The precise molecular mechanisms underlying these interactions are not completely understood. Further research is needed to elucidate the specific pathways linking diabetes to AD and to find potential therapeutic targets. Additionally, clinical studies are necessary to decide whether the effective management of diabetes can reduce the risk of developing AD or slow its progression in individuals with both conditions.

### 2.2. Controversies on the Pathological AD, Obesity, and T2D Relationships

This review cannot ignore the controversies that have arisen for so many decades in the relationships between metabolic diseases and AD based on different epidemiological, clinical, genetic, neuropathological, etc., studies. Important reviews of these types of studies have raised diverse types of doubts about the interpretation of the published results and the conclusions of the studies [37]. To reach a scientifically based conclusion, it is necessary to follow an objective study of the publications (and their conclusions) on the relationship between T2D and AD (or cognitive decline). We have reviewed research papers and reviews from important authors to reach informed conclusions about the relationships between T2D and AD.

For many decades, it has been proposed that T2D (and related pathological situations such as obesity, pre-diabetes, or “metabolic disease”) induces cognitive disorder and AD in humans. These conclusions have been confirmed by studies on AD and T2D models. This involves a main and direct action on neurons and glial cells. But other studies seem to indicate that the greatest damage to the brain is due to the direct interaction between the structure and function of the vascular vessels and the blood–brain barrier (BBB) [98,99]. That is, T2D would induce cerebrovascular dementia before a later-developing AD [78,100,101,102,103]. This would go against the concept that AD is a type 3 diabetes.

In a well-known review by Nelson et al. in 2009 [104], several problematic questions were raised about the relationship between T2D and AD. They considered eight reasons to affirm their relationship and another eight reasons to question it. This idea was shared in other reviews and works of that decade, even considering that T2D and AD were two different pathological entities that coincided in very (low?) specific cases. Studies conducted on experimental animal models seemed to confirm that a T2D-AD comorbidity exists. Special importance was placed on the results obtained with normal rodent models where T2D was induced with neurotoxins or transgenic models of AD, where neurodegeneration was accelerated with diabetogenic diets. In many cases, in the interpretation of the results shown, the identity of the pathogenic mechanisms in the joint pathology continues to be questioned. We consider extremely important the demonstration through transcriptomic and/or gene expression analysis, published in 2022–2022, which shows neuronal, glial, and vascular alterations in T2D patients with few dementing alterations. Bury et al., 2021 [105], in a study on six cases of T2D with minimal AD neuropathology (Braak 0-II) and without confounding pathologies or dementia, found significant dysregulation of several key signaling pathways associated with T2D, the cell cycle, and cellular senescence and also of AD-related genes (amyloid vein precursor protein and neprilysin) and down-regulation of mitochondrial respiratory electron transport. They also found alterations in gene expression in astrocytes and endothelial cells, all of them compatible with alterations in T2D and AD (astroglial reactions and alterations in the vascular endothelium/BBB). The alteration of these genes involved in pathogenic mechanisms “provides a potential point of intersection between T2D and AD neuropathology”, says the authors.

Neuropathological alterations are interpreted in quite diverse ways. For most authors, there are many causes of confusion ranging from the non-rigorous selection of pathological cases to the failure to compare each case with its clinical history (the duration of the T2D and AD disease, pre-diabetic phase, treatment phase and compliance, and so on). Long treatments with antidiabetic drugs can condition changes in the pathogenic course of AD [33] or induce changes in the presentation of the most characteristic signs of AD. The lower accumulation of amyloid plaques and neurofibrillary tangles, as well as the differences in the density and location of microglial cells in different cases of T2D-AD, could be due to these causes and not to a differential T2D and AD pathogenesis.

Currently, the idea that T2D and AD are two closely related pathologies due to common pathogenic mechanisms is considered to be the majority, as presented in the previous subchapter. Likewise, the consideration of AD as T3D is gaining followers.

The high incidence of cerebrovascular neuropathology or involving alterations of small vessels, always present, should not be ignored [99,103], nor should the existence of high stroke rates in T2D patients. This associated cerebrovascular pathology, which is not so far from AD neurodegeneration (there are actually many mixed AD-vascular dementia cases), would be another link between T2D and AD. Most of the brains from T2D-AD cases could show an initial/low degree image of AD since the coincidence of the two pathologies could accelerate the death of mixed T2D-AD clinically severe patients. Remembering the association between midlife vascular risk factors from different origins and brain amyloid deposition is a very frequent observation [98].

In many models of AD, T2D, and AD-T2D, the relationships between these two pathologies have been studied, trying to clarify whether vascular deterioration is predominant or not in the development of cognitive impairment. Cerebrovascular neuropathological differences exist between pre-diabetes and diabetes [106]. There seems to be an increase in AD pathology when pre-diabetes has induced vascular alterations [106]. In all cases, there are vascular alterations, and the authors give them greater or lesser importance according to quite subjective criteria. Cognitive dysfunction is attributed to vascular deterioration in studies on models. Takeda et al., 2010 [78] crossed AD transgenic mice with diabetic mice (ob/ob and NSY mice), producing different models, and analyzed their brain pathology. The induced diabetes in the AD basic model worsened cognitive dysfunction without an increase in brain amyloid-beta burden but showed intense cerebrovascular inflammation and severe amyloid angiopathy. High-fat diet feeding induced severe memory deficits in APP (+)-NSY mice without an increase in brain amyloid-beta load. In their conclusions, obesity and diabetes are considered a cause of cognitive dysfunction in the absence of accelerated beta-amyloid deposition. Moreover, the Alzheimer neuropathology seems to increase diabetes alterations in the single obese and T2D models. In a study by Niedowickz et al., 2014 [107], obesity and diabetes are presented as causes of cognitive dysfunction in the absence of accelerated β-amyloid deposition. However, in this study, the authors conclude that diabetes and/or obesity in these mice leads to a destabilization of the vasculature, leading to strokes, and that this, in turn, leads to a profound cognitive impairment, which is unlikely to be directly dependent on Aβ deposition. In other studies, the development of cognitive impairment is clearly associated with induced/accelerated amyloid and/or tau pathology [108,109].

## 3. Neuroglial Cells in the Healthy Brains and Those Affected by Neuroinflammatory Pathologies (AD and T2D)

### 3.1. Neuroglial Cells in the Healthy Young/Adult Brain

Neurons are the basic cells of the Central Nervous System (CNS). During the development of the CNS, diverse neuronal types are differentiated that make up different neuronal nuclei with differentiated functions. These neurons interconnect, forming neuronal circuits that will fulfill all the functions assigned to the CNS. But all these neurons need the collaboration of other accompanying cells, the neuroglial cells, which provide structural, metabolic, and regulatory support so that they can correctly fulfill their functions. These cells are neuroglial cells of different lines/strains that have different specific neuroregulatory functions and that act in a coordinated manner. Neurons are the cells of the organism that present the greatest adaptation capacities to conduct the distinct functions that they have to fulfill in the face of changes in their environment, but they constantly need the additional support and help of the different neuroglial cells that accompany them.

Since the pioneering studies of Cajal and his disciples and other contemporary researchers, different basic types of neuroglial (or glial) cells with different neuroprotective and neuroadaptive functions have been known as astrocytes, oligodendrocytes, and microglial cells [110,111,112,113,114,115,116,117,118,119]. Oligodendrocytes include the new pro-oligodendroglial cells or NG2+ cells described in recent years [120]. Each of these neuroglial types presents different subtypes with different phenotypes that have specific functions to collaborate in the function of neurons and maintain neuronal circuits. There is a permanent “crosstalk” between neurons and their accompanying neuroglial cells (and between the distinct types and subtypes of these cells) to achieve an adapted response at each moment and in each scenario [7,19,20,121,122,123,124].

An enormous number of “intercellular messengers” are used (cellular activators and inhibitors, pro-inflammatory and anti-inflammatory, neurotrophins, cytokines, chemokines, prostaglandins, metabolites, oxidants, antioxidants, and so on) to induce adaptive changes or collaborate in the correct functioning of each neuronal or neuroglial type [7,19,20,124,125,126,127,128,129,130,131]. Likewise, they “transfer” substances and mediators included in micro vesicles or exosomes [132,133,134,135,136,137,138,139,140,141,142]. All of these substances used, released into the environment, or transported within vesicles or exosomes can have different neurotoxic, neuroprotective, or ambivalent capacities (depending on the scenario in which neuronal or neuroglial responses should develop) [137,138,139,140]. Cellular communication or “crosstalk” between neurons and each of the different neuroglial types/subtypes (and vice versa) is constant, flexible, adaptable to circumstances, and modifiable by each cell type based on its state of adaptation or response [137,138,139,140,141,142].

In a very summary way, some important aspects of the three types of neuroglial cells should be remembered:

**Astrocytes** are CNS cells of neuroepithelial origin. Protoplasmic and fibrous morphological subtypes have been described over the years of their study with a wide variety of functions. New biochemical and molecular biology techniques (single-cell studies, transcriptomic and metabolomic analysis, etc. [143,144,145,146,147]) have revealed, through the expression of different genes, a high heterogeneity of astrocytes (astrocyte subtypes or astrocyte phenotypes) in relation to their location in the different cortical and hippocampal layers [148,149], as well as in both normal resting and abnormal degenerative situations (covered later). These morphofunctional subtypes can originate during CNS development or be induced by changes in the tissue environment and/or intrinsic astroglial adaptive mechanisms as part of adaptive, neuroreparative, neuroprotective, or neurotoxic astrocytic responses [19,20]. The laminar position, morphology, and gene expression profiles of cortical astrocytes are influenced by the time of birth from ventricular/subventricular progenitors [150]. Single-cell transcriptomic and epigenomic analysis identify candidate regulators of cell identity and distinguish the heterogeneity in neuroglial subsets.

The astrocytes not only configure a support network of the tissue architecture surrounding the neurons and the vascular network but regulate all the cerebrovascular functions [19,151]. These cells also maintain the homeostasis of the environment in which neuronal circuits develop but also participate in the development and maintenance of synapses, as well as in neurotransmission throughout the life of the individual functions [19,151,152,153,154,155]. These neuroglial cells are also involved in both neuronal glutamatergic transmission (the so-called neuro-astroglial tripartite synapses [155,156,157,158,159,160,161] as well as modulating cholinergic neurotransmission [162].

The vascular envelope gives rise to a key element of the CNS, the blood–brain barrier (BBB), which “isolates” the brain from many substances that can circulate through the blood and be harmful to neurons but allows, through astrocytes, the proper metabolite supply. Astrocytes can increase the uptake of glucose from the bloodstream and other metabolites and diffuse them into neurons through astrocyte networks to meet the functional needs of neurons. Astrocytes can accumulate glycogen (glycogen is exclusively localized in these cells in the brain) that can protect neurons in hypoglycemia [163].

Brain energy metabolism is based on metabolic cooperation between astrocytes and neurons [164,165,166,167]. This relationship is regulated by inducer factors acting on astroglial cells. Pro- and anti-inflammatory cytokines induce different metabolic phenotypes in astroglia [164,165,166,167,168]. VIP- and noradrenaline-induced glycogenolysis and the glutamate-stimulated aerobic glycolysis produce and release of lactate from astrocytes as an energy substrate for neurons (astrocyte–neuron lactate shunt [167]). Lactate production is necessary not only as an energy substrate but also as a signaling molecule for long-term memory maintenance and consolidation and for the regulation of the functions of dendritic spines [169,170,171]. It is important to note that lactate also stimulates the expression of synaptic plasticity- and neuroprotection-related genes such as Arc, Zif268, and BDNF [172,173]. Neurons also have an elevated level of glucose consumption through oxidative metabolism, which causes considerable amounts of reactive oxygen species that must be compensated by the production of antioxidant substances (especially by astrocytes), although in certain circumstances, they produce oxidative damage [174,175,176]. Oxidative stress has been commonly associated with other pathogenic mechanisms such as cellular senescence, mitochondrial dysfunctions, and other neurodegenerative processes [177,178,179,180].

**Oligodendrocytes** have been described in studies by Rio Hortega and Penfield [115,119] as protectors of neuronal axons due to their ability to form myelin sheaths. But there is a vast variety of subtypes specialized in exercising various support and regulation functions of neurons [181], including pro-oligodendrocytes (or NG2+ cells), which also exist in adult brains [182,183,184]. Oligodendrocytes also provide metabolic support to neurons. They accept glucose and convert it into lactate that they transfer to neurons. Lactate is released in peri-axonic spaces via monocarboxylate transporters (MCTs) [185] and taken up by neurons by other MCTs. They also release vesicles (EVs) that have gluconeogenesis-regulating enzymes that enhance neuronal metabolic activity [73]. There is a neuron–oligodendrocyte–astrocyte axis [186] that is key to supporting optimal neuronal function.

**Microglia cells** are considered the representatives of the immune system in the CNS, as well as those in charge of cutting cell debris that can be toxic. But, in addition, they have other important support functions for neurons, especially in the regulation of synapses [19,20,125,187,188] and in the adaptation of neurons to changes in the environment. Microglia play a key role in dynamically regulating neuronal activities through adaptive processes of synaptic pruning and phagocytosis [189,190,191]. The direct contact of microglia cells with neurons [192] and the constant intercommunication between microglia cells and neurons and other neuroglial cells [mediated by neurotransmitter and intercellular messengers (cytokines, chemokines, neurotrophins, etc.) acting on their specific receptors] helps support the optimal functioning of neural circuits [19,20,73,121,158,188,192,193,194]. The intercellular messengers, as well as other cellular-activating products, can be secreted or transported by vesicles or exosomes [195,196]. Direct neuron–microglia cell contacts are membrane appositions of microglial processes, rich in purinergic receptors that are activated by ATP released in the neuronal membranes, with selected areas of membranes of neurons, which have in their immediate proximity a cytoplasm that is locally enriched in mitochondria and the endoplasmic reticulum [192]. In these regions, microglia receive direct information about neuronal activity apart from that obtained through specific receptors for substances released by neurons. Microglia exert various effects on neurons and nervous tissue in general: neuroprotective, pro-inflammatory (beneficial in that they help drop harmful agents), anti-inflammatory (neutralize the harmful effects of inflammation and reversal of the inflammatory response when it is no longer necessary), antioxidant, and the maintenance of homeostasis. All of these effects are linked to genetic/functional changes suffered by these cells when they change to express different pro-inflammatory or anti-inflammatory phenotypes. Formerly, two subtypes of microglial cells, called M1 and M2 microglial subtypes, were considered, but now, authors prefer to refer to the expressed phenotype of each subtype of the microglial cell being studied or a description [197,198]. Similar to that described in the astroglia, innovative technologies currently allow us to have databases to type glial cells in different situations [199].

Microglia cells preferentially use glucose, but under conditions of decreased glucose levels, they can use other energy substrates (glutamine and fatty acids). Glucose is used via the oxidative phosphorylation pathway, but when resident microglial cells are activated under altered physiological conditions, there is a metabolic drive toward glycolysis. In these new conditions, the induced genes to transcript are those that cause the synthesis of cytokines (IL-1 Beta, IL-6, and TNF-alpha) [200].

In earlier paragraphs, the main defining characteristics of the different neuroglial types and their relationship with different physiological processes inherent to the normal functioning of the CNS have been presented (neuronal and neuroglial metabolism, the maintenance and proper functioning of neuronal circuits, and the maintenance of homeostasis). But currently, none of these processes have focused on a neuronal or neuroglial type that can be studied as an isolated mechanism since all of them are the result of the joint responses of neurons and all the accompanying glial cells. We must always keep in mind that neuroglial cells are very dynamic cellular elements that constantly respond to neuronal needs and the most adequate functioning of neuronal circuits. Each subtype of neuroglial cell adapts to changes in the local environmental situation or modifications of the close-related neurons to achieve the best functioning of neurons and neuronal circuits. In different neuroglial cells, subtle (or more pronounced in selected cases, as discussed below) morphofunctional and/or phenotypic changes occur to fulfill specific brain functions. These functions can be both basic and high-level. Among the first, we can mention the maintenance of homeostasis, the neurotransmission between certain neurons—the modern description of the “tripartite synapse” in which astrocytes have a capital role, or the permanent remodeling of synaptic complexes, especially responsible for microglia and pro-oligodendrocyte cells and the immune CNS defense of microglia. The high-level brain functions that the brain develops (learning, memory, recognition, etc.) also require glial adaptations in parallel with neuronal adaptations.

The most profound changes in neuroglial cells, which mainly occur in the face of very important alterations in the environment of the nervous tissue (such as those that occur in infections or metabolic diseases) or that are triggered by aberrant changes in the neurons that accompany/protect, are analyzed in the following subchapters.

### 3.2. Neuroglia in Brain Aging, AD, and T2D

#### 3.2.1. Reactive Neuroglial Cells in the Aging Brain and Brain Disorders. General Considerations

For many decades, most researchers have considered that all involutional/degenerative changes in the CNS were based on neuronal alterations (degeneration, dysfunction, and neuronal death). Currently, and based on studies from recent decades, this neuron-centric concept of brain diseases or disorders has changed, and gliopathology is considered to be as important, or more, than neuronal pathology in the strict sense. Firstly, diseases of neuroglial etiology have been described (due to genetic or induced causes). Secondly, it has been confirmed that there is no brain pathology in which pathological changes of the diverse types of neuroglia do not crucially intervene. Neuronal and neuronal circuit changes never occur isolated in the CNS without changes in neuroglial cells [19,20,32,73,122,123,124,125]. Pathological neuronal changes, or changes in the neuronal/neuroglial environment, start both degenerative and reparative processes (in the first instance, to compensate for the alterations that occur). Always, neuroglial cells have parallel adaptive changes (glioprotection mechanism) that tend to correct the neuronal environment or aberrant modifications of neurons or to eliminate the cytotoxic neurons produced by various neurotoxic/neurodegenerative agents. If these beneficial changes for the brain fail and/or the neuron involutional effects persist, inducing new alterations in the neurons and more extensive damage to the brain tissue, we enter a new scenario where dysfunctional/aberrant glial responses become the first actors in the brain pathology. The same occurs when there are aberrant glial responses due to both altered neuroglial genetic factors that affect the normal responses of some types of neuroglial cells or their functions or aberrant/persistent neuroglial activations. We then talk about gliopathogenesis [7,19,20].

The novel studies on gliopathogenesis should not make us forget that, in parallel to the neuron-centric studies of CNS diseases and disorders in the last century, important authors dedicated studies to the glial changes accompanying degenerated neurons [110,111,112,113,114,115,116,117,118,119].

In no way do neuroglial responses have to be considered “per se” pathological or detrimental; they are physiological responses. Its final effect on the brain (neurodegenerative and neuroprotective) will be the result of the conjunction of different neuronal and neuroglial responses within the context of the environment modified by toxins, metabolic alterations, infections, traumas, etc. [19,20,32,123,124,125].

Next, we want to highlight the main characteristics of neuroglial responses manifested in the brains of elderly patients or those suffering from AD, obesity, or T2D (the reason for this work), as well as other disorders or diseases that affect the CNS.

##### Astroglia

**Figure 2:**
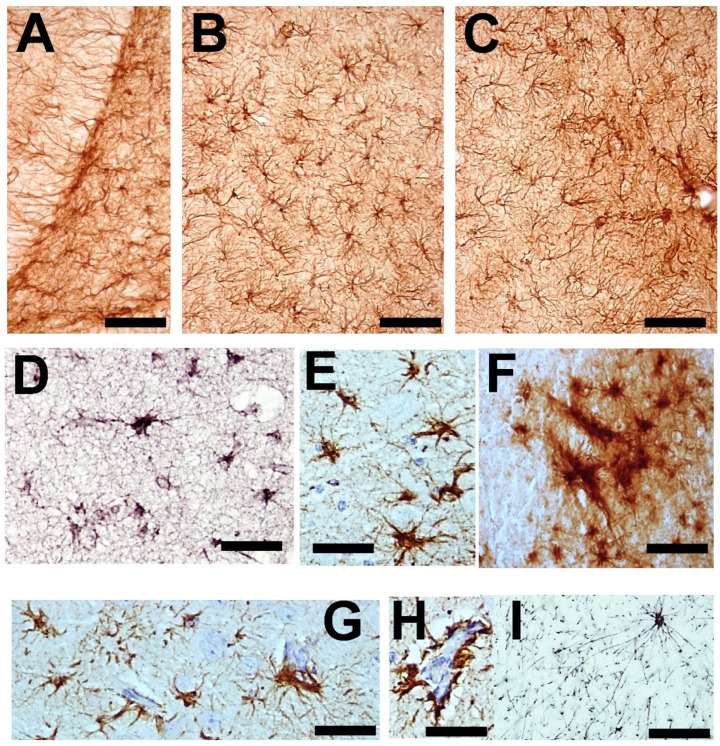
**Reactive astrocytes**. (**A**–**C**) Reactive astrocytes induced in hippocampus. (**A**,**B**) Reactive astrocytes induced by pilocarpine injection in rats. All astrocytes react simultaneously, maintaining their location and morphology ((**A**): dentate gyrus (DG); (**B**): DG hilus)). (**C**) Reactive astrocytes with different intensities and dislocation after injection of pro-inflammatory lipopolysaccharide (LPS). (**A**–**C**); GFAP immunostaining without contrast). (**D**) Reactive astrocytes in Brodman area 46 from an AD Braak V case. Reactive astrocytes with their complex network of fine processes (intensification of the GFAP-immunoreaction with Ni). (**E**) Reactive astrocytes in Brodman area 46, layer III, from an AD Braak V case (GFAP-immunoreaction, hematoxylin contrast). (**F**) Cluster of GFAP hyperreactive astrocytes (normal and hypertrophic sizes, including “velate” type) in the intersection of the granule cell layer and the white matter of the cerebellum of an AD case (GFAP immunostaining and hematoxylin contrast). (**G**) Reactive astocytes associated with a blood vessel (left) and a neuron bearing a prominent tangle. (**H**) Hypertrophic perivascular astroglial endings surrounding a blood vessel. (**G**,**H**), GFAP immunostaining with contrast of hematoxylin). (**I**) Brodmann area 46, brain of a 96-year-old man with no signs of cognitive decline. Astrocyte displays unusually long processes running through all layers of the cortex. Some astrocytes of this last type have also been observed in the cerebellar cortex. BAR (microns): (**A**,**D**,**F**,**G**) = 50; (**E**) = 40; (**H**) = 20; (**I**) = 75.

In response to aggressive agents (toxins and infections) or to abnormal changes in the tissue environment or in the neurons that accompany (produced by lesions, aging, and neurodegenerative processes), astroglial cells react [19,20,111,112,151,201,202,203]. Diverse types of responses have been described in different astroglial elements (Figure 2). In the classical definition of (astro)gliosis, hypertrophy coexists with cellular hyperplasia. Moreover, involutive processes (named klasmodendrosis or klasmatodendrosis) are present in other cells [7,110,145,146,204,205,206,207]. Reactive astrocytes undergo phenotypic changes. Quite different subtypes have been detected but are conventionally grouped into two functional subtypes of opposite neuronal action: neurotoxic (A1) or neuroprotective (A2) [19,20,122,151,208,209,210,211]. Gene transcriptome analyses of reactive astrocytes show preferentially expressed genes in these two types of reactive elements, such as complement-3 (C3), guanine nucleotide-binding protein 2 (GBP2), and Serpin1 in A1 reactive astrocytes and S100 (calcium-binding protein A10), pentraxin-3 (PTX3), and S1Pr3 in A2 reactive astrocytes. Many other genes (more than 150) were preferentially expressed in induced A1 or A2 astrocytes [212]. In pathological studies, the most commonly used specific markers are C3 for A1 astrocytes and S100a10 or PTX3 for A2 astrocytes. A1 reactive astrocytes are mainly induced by reactive microglia that produce pro-inflammatory cytokines such (TNF-α, IL-lα, and IL-18), as well as other pro-inflammatory molecules such as complement C1 subtypes, NO, oxidants, and the inflammatory effector cytokine processed by NLRP3 inflammasomes. These molecules are, in most cases, repressors of A2 astrocytes [64,70,202,203]. Studies on triple-knockout mice (IL-lα, TNFα, and C1qa) have shown that A1 reactivity decreased after neuroinflammatory induction. Moreover, these reactive cells are not generated in animals devoid of microglia, such is the case of the CSFR-knockout mice [202,203,213,214]. Aberrant and/or persistent activation of normal functional astrocytes provokes phenotypic expression of A1 subtypes, which in turn initiate the neuroinflammatory reaction acting on the microglia [7,19,209,210]. A1 astrocytes produce different neurotoxins, such D-serine, NO, and pro-inflammatory cytokines. In this way, A1 astrocytes can induce neurodegeneration and neuronal apoptosis as well as neurodegenerative alterations in oligodendrocytes (including pro-oligodendrocytes) and microglial cells [215]. It could be thought that these generated A1 astrocytes initiate and progressively reactivate the neuroinflammatory process until irreversible neurodegeneration occurs. This is true in many cases, but it must be taken into account that a neuroinflammatory mechanism can also be set in motion to try to recover functional normality after the elimination of the unrecoverable neurons [216,217,218]. TGF-β and FGF down-regulated genes related to the A1 phenotype [216,217].

A2 astrocytes are mainly induced by anti-inflammatory cytokines (IL-1β and IL10) secreted by microglia of the M2 phenotype [219] and a chemokine, prokineticin 2 (PK2), which is preferent produced in neurons [215,220,221]. A2 astrocytes are key elements in the defense against neuroinflammation originating in microglial cells [215]. Moreover, they have important roles in neuroprotection and neurorepair, as well as the maintenance of the neuronal circuits regulating synaptogenesis. These functions are mediated by the production of neurotrophic factors, anti-inflammatory cytokines (L6 and IL10), and thrombospondins.

A1 and A2 astrocytes observed in different physiological and pathological scenarios, as well as the production of neurotoxic or neuroprotective substances, are interpreted in very different manners. Consequently, important controversies are created that are difficult to resolve and that lead to delays in possible treatments because the beneficial or toxic effect of many of the observed phenotypes is doubted [7,19]. Given this, in the review of Escartin et al. in 2021 [211] on the reactive astrocytes, a consensus is proposed to define subtypes. The authors propose that in studies on astroglia, the subtypes studied should be defined with great precision, including morphological and functional data, both local and general (morphology, location, genetics, RNA expression, neuronal, and glial effects). In this way, their involvement could be better understood in glioprotection and gliopathology. Astrocyte-derived extracellular vesicles are novel regulators in neurodegenerative disease progression and a potential pathway for therapy [142,222,223,224,225,226]. It should be noted that new studies in molecular biology and genomics, using an analysis of individual cells representative of the state and reactivity of each subtype of astroglial cells in each pathological scenario [144,149,227,228,229], provide and will continue to provide in the coming years a strong and indisputable basis for the neuropathological course of neurodegenerative diseases and their effective treatment.

###### Oligodendroglia

The reactive responses of oligodendroglia are the least studied in the physiology and pathology of the CNS. On this topic, the studies conducted can be differentiated into two distinct aspects: the responses to attacks that concern problems in myelination (demyelinating diseases and stroke) and the involvement of pro-oligodendrocytes in general disorders affecting the CNS [230,231]. Regarding the first aspect, it has been shown that the affected oligodendrocytes emit signals (directly secreting cytokines and chemokines or releasing vesicles loaded with intercellular messengers) tending to activate other neuroglial cells [230,231,232]. The aim of these induced responses is twofold. On the one hand, they activate the phagocytosis of myelin remains by astrocytes, as well as launch the neuroinflammatory response (which can subsequently lead to a reparative anti-neuroinflammatory response). On the other hand, they activate pro-oligodendrocytes (OPCs) so that they are induced to transform into mature myelinating oligodendrocytes and thus can remyelinate damaged axons in an induced degenerative process or in a neurodegenerative disease. In the latter case, response failure is practically certain, but it continues to be studied with great insistence since it may be the key to therapies in diseases such as amyotrophic lateral sclerosis. Remyelination, the restoration of new myelin sheaths to demyelinated axons, is not performed by pre-existing mature oligodendrocytes [232] but involves, in most cases, the generation of new mature oligodendrocytes from the adult, the quiescent OPC pool distributed throughout the CNS [230,231,232,233,234]. 

Regarding the second aspect, the reactivity of resident OPCs, widely represented in all regions of the CNS, suggests that these cells have foremost importance in both glioprotection and gliopathogenesis of the CNS. OPCs are not a uniform population; in different regions/subregions, the cells show different behaviors or functions. The responsiveness to growth factors or mitogens is region-specific. OPCs in the white matter respond to the platelet-derived growth factor, a fact not observed in the gray matter [235]. Moreover, their capacities to differentiate when transplanted into other CNS areas are very different depending on environmental conditions [236,237]. There are doubts about whether the different properties observed in different subgroups of OPCs are the manifestation of the existence of different subtypes/phenotypes, of different functional states of the same cells, or of programmed changes in the progression of their lineage [181]. The different phenotypes described do not correlate in an indisputable way with certain regions of the CNS, with certain pathological states, or with age [236,237,238,239,240]. OPCs appear to proliferate and enter damaged areas of the CNS; the greatest accumulations have been observed in areas with neurodegenerative lesions [238,239,240]. A subset of OPCs that do not express Olig2 during development can be increased in the adult by brain injuries [233]. In different studies, senescent pro-oligodendrocytes appear to increase in regions with greater neuropathological alterations, both in humans and in experimental models of AD. In these studies, the use of senolytics can, in some cases, improve cognitive involution and delay the progression of neuropathology [182,241].

###### Microglia

**Figure 3:**
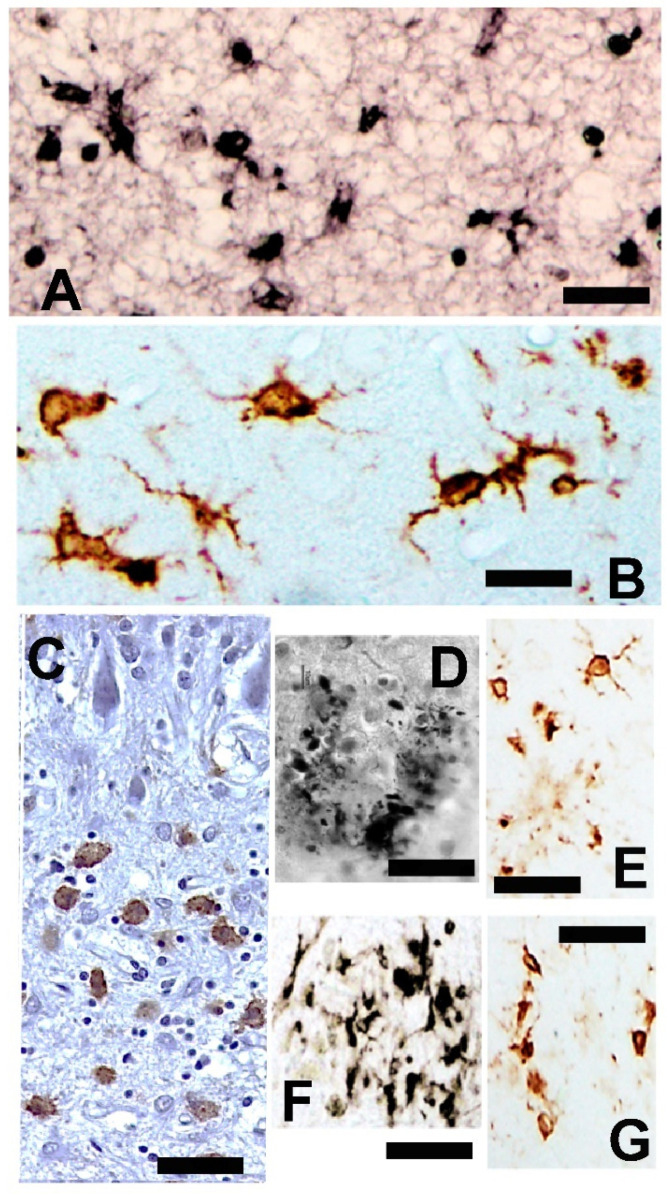
**Microglial reactive cells**. Microglial reactive cells of different morphological types (rounded with few processes, elongated or fusiform with long/medium processes, each of them more or less branched). (**A**) Different morphological subtypes with different processes of distinct size forming a complex plexus in the neuropil of an area of the Brodman 46 region from an AD Braak V brain. Staining with LN3 intensified with Ni. (**B**) Neuropil of hypercaloric treated AD mice, frontoparietal cortex. Hypertrophic branched elements. Iba-1 immunostaining without contrast. (**C**) Hippocampal polymorphic sub-CA3 layer in a case of Braak IV human AD showing highly hypertrophic phagocytic round microglial cells. Iba-1 immunostaining with hematoxylin contrast AD case, Hippocampus CA 3 region. (**D**) Amyloid plaque invaded peripherally by microglial cells (Bielchowsky silver-impregnation technique); Brodman area 46 from an AD Braak V case. (**E**) Atrophic microglial cells related to a plaque without reactive microglial cells The same case of D. (**F**) Large amyloid plaque invaded by microglial cells in a case of diabetogenic treated APP-PS1 AD model. Iba-1 immunostaining with hematoxylin contrast and Ni reinforcement. (**G**) Microglial cells forming a crown not closely related to an amyloid plaque in the same case. BAR (microns): (**A**–**C**) = 50; (**D**,**F**) = 65; (**E**,**G**) = 60.

Many types of reactive microglial cells (using different alternative names such as “activated” or “hypertrophic”) have been described in aging, neurodegenerative, and/or brain disorders [242,243]. Microglia exhibit regional-, age-, and neurodegenerative-dependent phenotypes [244,245,246], although their exact role in each case is not yet fully clarified. Microglial cells are a heterogeneous and dynamic population of mesodermal cells in the CNS. In the adult brain, their coupled proliferation and apoptosis maintain a rapid turnover of microglia [247] regardless of the phenotypic changes that many elements undergo to fulfill their functions. Morphologically, subsets of activated microglia present an increased number of amoeboid forms, but other subsets are characterized by a decreased arborization of the peripheral prolongations (not excluding hypertrophic forms) [248] (Figure 3). 

Reactive microglial cells are commonly distinguished in research studies by the overexpression of a low number of specific molecules (human leukocyte antigen D related (HLADR) and clusters of differentiation (CD) molecules such as CD40, CD45, and CD68 [249,250]), but more detailed studies in different biomedical fields show a very high number of new phenotypes. Reactive microglial cells can be classified into two main opposite states or phenotype expressions regarding the pro-inflammatory/neurotoxic substance secretion (cytokines such as IL-1α, IL-1, IL-6, IL-12, IL-15, IL-17; monocyte chemoattractant protein-1 (MCP-1); and TNF-α; as well as reactive oxygen and nitrogen (ROS and NOS) species) or anti-inflammatory/neuroprotective secretion (cytokines such as IL-10 and IL-4 and transforming growth factor beta (TGFbeta)) [251].

M1 and M2 main subtypes (pro-inflammatory and anti-inflammatory phenotypes) have been considered over the years [7,19] to be closely related to the induction and expression of, respectively, A1 or A2 reactive astrocytes. However, new interpretations of microglial responses are gaining ground in this field of research. Ransohoff 2016 [197] already questioned the drastic division into these two functional subtypes. The existence of M1 or M2 cell subclasses in any brain region has not been able to be unquestionably defined. Nor can it be demonstrated that any phase of the neurodegenerative process correlates with certain M1 or M2 cell phenotypes [244,245,251,252]. The new markers that are being proposed to define microglial cell subgroups show a great difference in expression in different brain areas [252]. The behavior of microglial cells is extremely complex, and the expressions of their different genes related to the secretion of pro- or anti-inflammatory substances in certain cells can be modified according to pathological circumstances that should also be defined more precisely to understand microglial responses. It is necessary to resort to new studies of genomics, epigenomics, transcriptomics, etc., with the development of new technologies to characterize patterns of functional behavior of microglial cells that must be regulated to obtain the corrective benefits of the microglial response, both in the phagocytic aspect and in the production and release of factors that induce/modify neuronal responses and those of other glial cells or the tissue environment. The existing encyclopedia of glial cells must be constantly updated and expanded [199]. The use of the M1 or M2 nomenclature should currently be understood more as a definition of a functional response (pro-/anti-inflammatory) than as a definition of specific cells. The relationship of microglial cells with neurons or neuroglia cells is established both with the secretion of substances, such as vesicles, and exosomes that transport large molecules and RNA to greater distances [140,196]. Although there are all these controversies about functional subtypes and behavioral variations of microglial cells, there is a total consensus on the absolutely critical participation of microglial responses in neurodegenerative diseases, as demonstrated by their inclusion in all neuropathological cascades that lead to the development of dementia [7,19,253].

Microglial involutive changes have been described in the human brain in aging and in neurodegenerative diseases [254]. The term of “dystrophy” includes different morphological abnormalities that affect peripheral microglial extensions (spheroid swellings, loss of collateral branches, beaded or tortuous processes, and fragmentation of the extensions). The term “cytorrhexis” has been proposed to specifically describe the cytoplasmic fragmentation observed in microglial cells [254]. The involution of microglia has been correlated with neurofibrillary degeneration, considering that neurodegeneration is accelerated after age-related microglial involution. 

As was described, new microglial cells can originate from macrophages, which enter the brain parenchyma through Virchow–Robin spaces [117]. The existence of invasive microglia in neurodegenerative diseases has been questioned in recent years [255,256]; although it is accepted in other neuropathological processes such as stroke [257], multiple sclerosis [258], and epilepsy [259], it is now denied that this occurs in AD [260]. Until recently, resident microglial cells could not be distinguished from microglial cells originating from invading monocytes [255,256], but it has now been shown that microglial cells associated with amyloid plaques and degenerating neurons originate from resident microglial cells [260]. However, monocyte–microglia cooperation could be effective in areas with a disrupted BBB.

#### 3.2.2. Neuroglia in the Aging Brain

It is essential to know the state/situation of the neuroglia during normal brain aging to be able to correctly evaluate the changes induced in the Alzheimer’s brain, the brain of a type 2 diabetes patient, or another who suffers from AD and type 2 diabetes. These pathologies occur, with exceptions, at advanced ages. Although it seems easy to analyze the neuroglial changes produced in old age, there is still a lot of controversy on this topic. Many of the studies published on this item have been conducted on people classified by age and the criterion dementia/non-dementia without considering other concurrent pathologies. There is no clearly accepted limit for considering a brain to be young or old. The criterion used to differentiate early onset AD from late-onset AD (65 years of age) could be used. It is not a strictly scientific criterion, but differential characteristics are shown in each group related to their own genetic and neuropathological traits.

The studies conducted since the beginning of neuroscience seem to indicate that there is no single pattern of senile involution applicable to all regions of the CNS and that each of them must be studied in detail.

In a general way, the neuropathological descriptions (at the level of optical microscopy) of the brains of healthy elderly individuals (who do not suffer from neurodegenerative or systemic diseases) could be said to be among the descriptions of young, healthy humans and those of people with Alzheimer’s or other neurodegenerative diseases. Studies in senile animals (both at the level of optical and electron microscopy) have confirmed the findings in humans and have broadened their interpretation with the help of biochemical and molecular biology studies carried out in parallel. Today, with the help of new technologies for genome analysis, transcriptomics, and metabolomics using single-cell technology, it can be said that we already have some basic cellular patterns to differentiate healthy adult and senile human brains, as well as to differentiate the latter from those who have suffered from some neurodegenerative pathology. Regardless of the fact that in the whole brain and/or different regions of the CNS, changes in their neurons associated with aging are being defined and used to characterize the involution of the brain/each region [261], it is becoming clear that there are important changes in neuroglial cells related to age. Signatures of different subtypes of astroglial [262], oligodendroglial [263], and microglial cells [264,265,266] have been published in recent years. This has opened new avenues to understand in depth the new scenarios that are appearing in each region of the CNS where neurons and glial cells coexist throughout life, as well as the pathogenic mechanisms and their possible therapeutic correction [267].

Healthy/normal senile brains are characterized by not showing (or showing in very low density) neuropathological alterations characteristic of neurodegenerative diseases (diffuse amyloid deposits or plaques and dystrophic neurites or neurofibrillary tangles typical of AD, accumulations of phospho-tau in tauopathies, etc.). Noticeable with morphological techniques, as general characteristic features of aged brains, a significant accumulation of lipofuscin in neurons, gliosis (astrogliosis and microgliosis in particular), and signs of demyelination were described. Statistical studies can find decreases in neurons, glial cells, and/or synapses in different regions of the CNS. More specialized studies, with modern immunohistochemical and molecular biology techniques, show the above-mentioned major changes in gene expression that lead to the manifestation of new phenotypes in neuroglial cells. A wide range of astroglial, oligodendroglial (including OPCs), and microglial cell subtypes have been described in this way. It has been observed that in different regions of the CNS of man and different mammals, there is a wide variety of neuroglial cells of each lineage that could be classified into various “subclasses” or “subtypes”: normal, reactive (with diverse phenotypes; generically classifiable into two antagonistic groups, pro-inflammatory or anti-inflammatory), senescent [268], or atrophic-degenerative. These glial changes, or new phenotypes, are especially significant in the production of extracellular messengers that induce neuroprotective or neurotoxic changes (that is, mediators of glioprotection or gliotoxicity). On many occasions, these changes in aging have been less valued than those that occur in neurodegenerative diseases. Biochemical and molecular studies are essential to understand the functional importance of neuroglial changes. In the senile brain, there must exist (we can say, without a doubt, that it exists) a new scenario of neuronal–neuroglial relationships where an attempt is made to maintain neuronal functions and neuronal circuits and compensate for the losses caused by age. Studies on centenarians show the great capacity for resilience of the CNS [269].

The variety of subclasses and phenotypes of each neuroglial type that is detected in the brain during aging makes it difficult to both classify them and define characteristic profiles of astrocyte, oligodendrocyte, or microglial cells at this stage of life. It is considered that “senescent” cells (neuronal or glial, characterized by expressing distinctive proteins such as beta-galactosidase) are characteristic of CNS aging and that this new aged neuronal–neuroglial scenario is basic for developing new neurodegenerative processes [268]. This is the truth, but not the whole truth. It is true that senescent cells are present in the aged brain (or in various regions of it), but it is also true that senescent cells are immersed in a plethora of different subclasses/phenotypes of other reactive or no-reactive elements of each neuroglial type. Therefore, different messengers secreted by the various neuroglial subsets can trigger different pathogenic mechanisms in neurons. It is true that senescent cells functionally are characterized by not being able to carry out the physiological functions of normal cells. In the case of neuroglial cells, they cannot perform the specific tasks of each type/subtype (neuroprotection/neurorepair, immune, antioxidant, or anti-inflammatory responses; phagocytosis), leading to an involution of nervous tissue. But it is true that this negative effect can be counterbalanced by the neuroprotective/neuroreparative effects of subsets of neuroglial cells that protect the involution of the CNS [270,271,272,273,274,275,276]. Moreover, diet and exercise can help to fight against brain aging involution [277,278].

We would like to highlight selected characteristics of neuroglial cells during aging.

##### Astroglia

**Figure 4:**
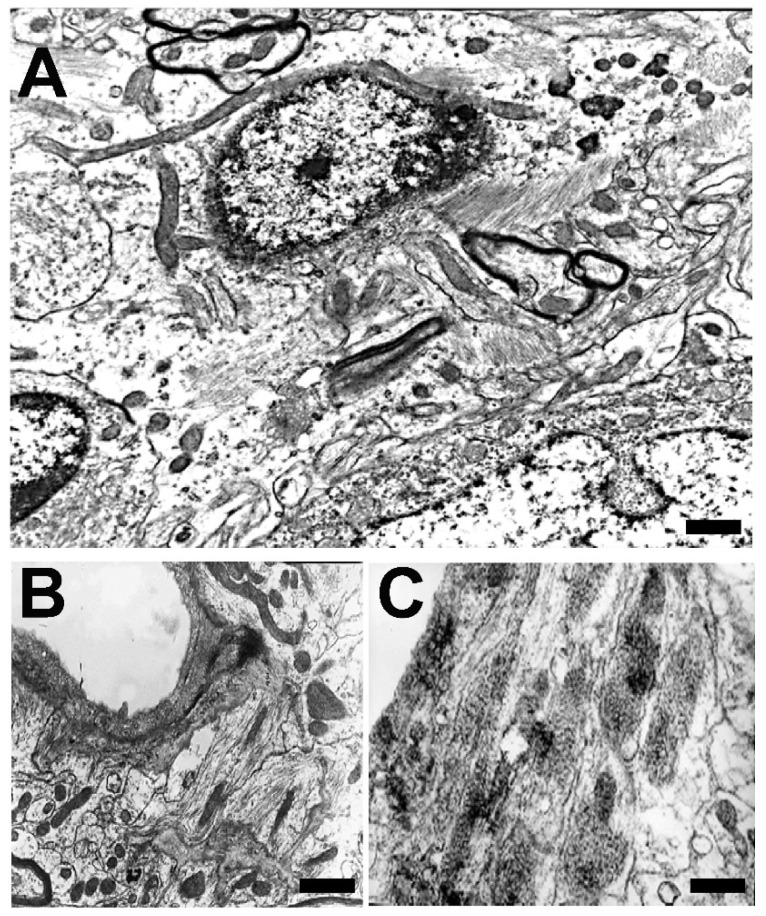
**Electron microscopy images of reactive astrocytes**. Granular layer of the cerebellum of 36-month-old rat. (**A)** Astrocyte showing numerous thick fascicles of gliofibrils and pleomorphic dense bodies, highly altered mitochondria of quite different sizes (hypertrophied and/or very long, or very small); dense bodies; concentric lamellae; and encapsulated small, round, pyknotic masses or degenerated axonal elements. (**B**) Hypertrophic perivascular astroglial endings surrounding a vessel with an abundance of inter-astroglia junctions. The blood vessel shows thickening of the endothelium and the basement membrane. This can negatively affect the BBB. (**B**,**C**) Different layers of hypertrophic perivascular astroglial endings surrounding blood vessels. Section of the glial processes normal to the gliofibril bundles. BAR (microns): (**A**) = 1.7; (**B**) = 3; (**C**) = 1.6.

Astroglia present a great diversity of morpho-histochemical forms (Figure 4), as well as reactive or involutive phenotypes (including senescent cells) [279,280,281,282,283,284]. For many authors, the hypertrophic/hyperreactive forms of the usual normal astrocytes and/or the increase of reactive astrocyte markers are prominent (both in the cell body, its perineuronal and/or perivascular processes, or long extensions that penetrate areas not reached by normal astroglial cells—Figure 2). Generally, hypertrophy/hyperreactivity is observed distributed randomly in areas of greater or lesser extension in a heterogeneous manner and affecting various astroglial elements (Figure 4). Special attention has been paid to these astrocytes that are located more or less close to dystrophic neurons, altered synaptic complexes, and altered blood vessels (Figure 4).

For other authors, the most important alteration is the morphological involution of the normal development field of the prolongations in the neuropil [125,281] or the increased presence of atrophic/involutive elements (produced by klasmatodedrosis or functional asthenia) [7,20,281]. Excessive astrogliosis, considered a distinctive sign of aging in some studies, seems to be not really so evident: it is currently believed that there is a certain maintenance of the number of astrocytes throughout life, but their functionality may be changed in many ways. Only a minimal to high degree of hyperplasia has been observed in several areas, such as in the Purkinje cell layer of the cerebellum [204], but without reactive manifestation of GFAP. Hypertrophic and hypertrophic/hyperreactive astrocytes show both a wide variety of astroglial markers (compatible with normal subtypes, A1 and A2) and a great diversity of the level of expression of these markers [281,282,283,284,285]. Atrophic astrocytes in aged marmosets present tau hyperphosphorylation, RNA oxidation, and DNA fragmentation [286].

To the astrocytes present in the aged brain, important studies have been attributed to many alterations observed at this stage of life in the CNS and, secondarily, in peripheral tissues: changes in cholesterol, carbohydrate metabolism, and protein catabolism [44,280,287,288]; alterations in immune responses and the development of a low/intermediate-grade inflammatory state (CNS inflammaging) [282]; the development or acceleration of cognitive disorders and neurodegenerative diseases or systemic diseases [280,281,283,284]; and alterations in selected receptors (adenosine) and factors (insulin-like growth factor (IGF1) and hypoxia-inducible factor 1α (HIF1α-)) [289].

A prominent issue is that, in studies on CNS aging, significant alterations have been found in the mitochondria of astrocytes [290,291,292,293,294]. Many authors point out that these changes (consubstantial to each individual or induced during his/her life) are transcendent both in causing unhealthy aging and in promoting cognitive problems or neurodegenerative diseases. Electron microscopic studies of senile animals show hypertrophic senile astrocytes with the accumulation of gliofibril bundles and mitochondria of vastly different types, from highly hypertrophic or dystrophic to very small forms. These mitochondrial forms can be interpreted as dysfunctional or difficult to reuse (by mitophagy failure) in the first case or as attempts at reparative mitogenesis in the second case (Figure 4). Another prominent issue is the involvement of astrocytes in the senile brain as basic components of the blood–brain barrier (BBB) [295,296]. Biochemical and functional studies show that in aging, there is a functional disruption of the BBB (including endothelial cells and the basal membrane, as well as the astrocytic envelope) that causes a deterioration of the brain, since there is an increase in the permeability of cytotoxic substances to the brain [297,298,299,300,301,302,303,304]. However, morphological studies in humans and in senile animals (at the level of optical and electron microscopy) (Figure 2 and Figure 4) show the existence of reinforced envelopes of astroglial extensions and hypertrophy of endothelial cells and the basal membrane, all of which suggest vascular sclerosis. BBB dysfunction and vascular sclerosis seem to coincide in brain aging, with astroglia mainly responsible to a significant extent. The deterioration/dysfunction of the blood–brain barrier (BBB) increases the passage of cytotoxic substances into the brain from different organs that may be undergoing pathological alterations. All of this can help the development or accelerate the pathogenic course of many pathologies such as T2D and AD. Alterations in the glymphatic system in aging have also been observed, inducing problems in the elimination of toxic brain products [298].

##### Oligodrendroglia

Oligodendroglia (from various subtypes of pro-oligodendrocytes/oligodendrocyte precursor cells (OPCs) to diverse myelinating oligodendrocytes (MOL)) are also important in brain aging. Aged alterations are observed in both myelinating and non-myelinating oligodendrocytes [299,300,301]. In some cases, the alterations can be observed using morpho-histochemical techniques, but in most cases, it is necessary to resort to biochemical, molecular biology, or genetic studies. Morpho-histochemical changes (low-inclusion bodies) are only partially indicative of the functional alterations that are manifested to a considerable extent by molecular studies. Myelinating oligodendrocytes undergo aging alterations that are often accompanied by degenerative changes to the myelin sheath and a decrease in white matter. White matter loss and myelin damage are strongly associated with cognitive decline (not clinical Alzheimer’s). These changes are considered induced by lifelong oxidative stress that damages the myelin sheath [301]. The number of subtypes of MOLs is maintained in senile age (from the initial/immature myelinating types to the mature ones). The total numbers remain more or less maintained in aging, although they vary in the different regions of the brain (decreasing in the frontal cortex) [299]. The most differentiated MOLs (mature OLs) decrease with age and need to be replaced by new elements from OPCs that differentiate into mature OLs passing through different stages of differentiation. If the process is completed, there will be no functional alteration of the brain, but if it does not reach full repair, there will be. Recently, a new subtype prior to mature oligodendrocytes has been described that, if it involutes, gives rise to progressive demyelination [302]. The remyelination process is regulated by involuted/demyelinated neurons, astrocytes, and microglial cells [303,304,305]. It is important to highlight that activated microglia in the aged brain release inflammatory factors that can suppress OPC differentiation [306].

OPCs are a large population of macroglia cells comprising various functional subtypes with distinct phenotypes [281,307]. The effects of OPCs are not restricted to being the reservoir of new cells producing mature OLs. OPCs have a wide range of protective functions of the neuronal environment, neurons, other types of neuroglia, and the BBB [307]. They also have phagocytic capacities [308]. These functions have been discovered in recent years and are becoming increasingly clear. However, it is also being discovered that many of them are neurotoxic and pro-inflammatory [307]. OPCs increase with age. The alterations in pro-oligodendrocytes are very variable in different regions of the senile brain.

Moreover, many of the elements of these subtypes/phenotypes may have new functions, opposite in many cases (neuroprotective or neurodegenerative). Many changes may be neuroprotective, but there is little information on this subject. Most of the described reactive OPCs are neurotoxic/neurodegenerative in nature; they can be considered senescent cells [306]. These kinds of cells are susceptible to being eliminated with senolytics (a combination of dasatinib and quercetin acting on these and other senescent cells, such as endothelial senescent cells [309].

##### Microglia

Microglia are the neuroglial macrophage population of the brain, dedicated to supporting the CNS environment and protecting it from endogenous and exogenous insults. Microgliosis has been described in the senile brain, with different interpretations, neuroprotective or neurotoxic [310,311,312,313]. Nonetheless, their normal functions decline with age and, therefore, it has been considered for many years as the main cause of brain aging functional decline. Considering that microglia have a wide range of physiological functions in the CNS, as mentioned in Section 3.2.1, it is pertinent to ask which of these functions may be affected in the brain during aging. In a review by Pannese, 2021 [299], on changes in neuroglia in aging mammals, it is noted that changes in microglia function affect proliferation in the response to injury, motility of cell processes, ability to migrate to sites of neural injury, and phagocytic and autophagic capabilities. That is, all of its functions. Different mechanisms govern microglial states, and the new scenario presented by the aging brain can induce functional microglial changes [314,315,316]. Compatible with this functional analysis of microglia in brain aging, a review by Antignano et al., 2023 [317] on the results of microglial studies using transcriptomic and genetic techniques in tissue or isolated cells shows the existence of different phenotypes that are characterized by the overexpression of a set of genes that are activated to carry out certain functions, both beneficial and detrimental to nervous tissue. Microglia lose their homeostatic molecular signature. Diverse microglia subpopulations and cellular states in aging and disease have been described: “disease associated microglia” (DAM), “neurodegenerative microglia” (MGnD), “dysfunctional microglia”, “lipid-droplet-accumulating microglia” (LdaM), and “protective phagocytic microglia” [264,318,319,320,321]. Different subsets, or phenotypes, of microglia already exist in young adults, but they expand in aging and even more in neurodegeneration. Except for the last group, all of them, although different in the induced functions and the messengers produced (cytokines, free radicals) and dysfunctional lysosomal deposit production indicative of impaired phagocytosis, have a high cytotoxic component (pro-inflammatory and pro-oxidant) [273,322]. Microglial elements of these groups trigger direct or indirect (by microglia-derived extracellular vesicles) age-related changes and neurodegeneration upon DNA damage [323]. Only the elements of the LdaM group can be easily detected with morpho-histochemical techniques.

Regarding this topic, there is a high degree of controversy about the classic classification of modified microglial elements (reactive, senescent, dysfunctional, phagocytic, etc.) [310,324]. An important number of authors consider “senescent”, although they do not meet all the requirements, all the modified cells observed in the aged brain and in neurodegenerative diseases. Other authors differentiate “senescent” cells (true, with a differentiated molecular signature) and “reactive cells” (of distinct types, with different signatures) [324,325,326,327,328,329,330]. Sex differences in microglia function in aged rats have been observed [331,332,333]. This fact can underlie vulnerability to cognitive decline [331].

**Figure 5:**
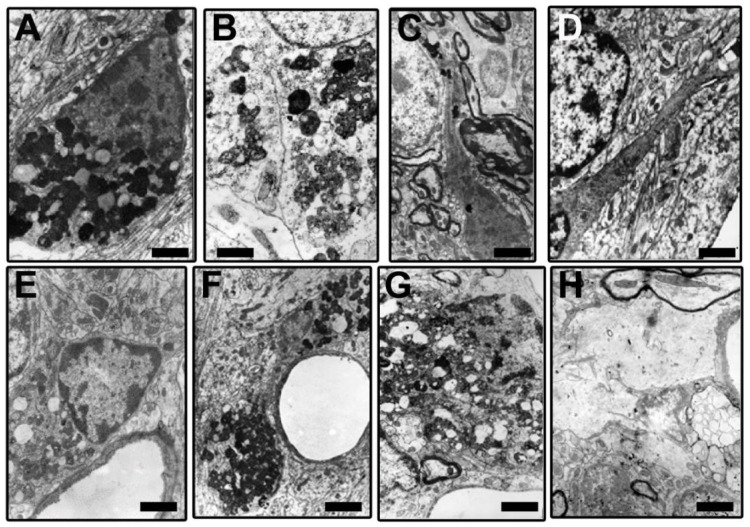
**Electron microscopy images of reactive microglial cells**. Cerebellum of 36-month-old rat. (**A**) Molecular layer. Microglial cell fully loaded with phagocytosed dense bodies. (**B**) Granule cell layer. Two reactive microglial cells in close contact. (**C**,**D**) Microglial reactive cells presenting a long hypertrophic process running through the neuropil and showing some phagosomes. (**E**–**H**) Microglial reactive cells associated with blood vessels with phagocytic characteristics. The vessels are dilated, and their walls (endothelium, basement membrane, and astroglial envelope) may be thickened. In some cases, microglial processes appear “empty” of subcellular debris (**H**) These apparently empty formations can reach large sizes. Several authors point out that some of them may also belong to astrocytic cells and degenerate in the empty perivascular spaces observed at the optical microscopy level, especially in degenerative diseases (T2D and AD). BAR (microns): (**A**,**B**,**E**,**G**) = 10; (**C**,**D**,**F**) = 20; (**H**) = 3.

Morpho-histochemicaly, it is observed that there is a wide variety of forms, much greater than those observed in young adults, although elongated or rounded forms with medium-sized and sparsely branched extensions may predominate, which is compatible with reactive elements. In the same areas, there are elements without extensions (of normal size or hypertrophic) suggestive of phagocytosis, as well as other hypertrophic elements with large polar extensions of fine caliber and branched or extensions with a tortuous course and with varicosities. Heterogeneous inclusions, including lipofuscin granules of diverse types, accumulate with age in microglia (Figure 5). In old age, nearly all microglia contain perikaryal inclusions, being the cells near the blood vessels (Figure 5).

Many elements are hyperreactive against various microglial markers. In some areas of various brain regions, there is a reaction homogeneity among the elements, but in others, the predominant pattern is of great heterogeneity [244,334]. Microglia numbers vary markedly with brain regions and species [299]. Several studies have shown a slight increase in microglia, especially in the cerebral cortex or cerebellum, and correlated with advancing age [our cerebellum], but others have not [299]. But contrary to widely held beliefs, it cannot be said that microgliosis is a characteristic of the aging brain.

The relationships of microglial cells with systemic cells of mesodermal origin (border brain-associeted or systemic) seem to increase [335], a phenomenon that can have beneficial effects but that also increases to increase the pathogenic course of many diseases (T2D and AD) [336]. 

Emerging roles of microglia in blood–brain barrier integrity in aging and neurodegeneration have been described in a recent study [337].

**Figure 6:**
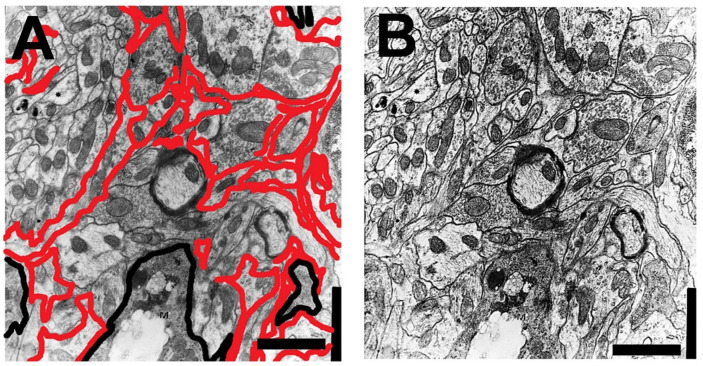
Electron microscopy images of hypertrophic astrocyte and microglial cell extensions in the neuropil of the cerebral cortex in senility. A 30-month-old rat. The astrocyte extensions (red in the left image) that surround the neuronal somas, dendrites, and axons are thicker and show constant variations in diameter. The microglial extensions (black in the left image) are more difficult to observe; but varicosities as is presented in the bottom, are observed in some cases. Microglial bodies with lysosomes, vesicles, and residual bodies, such as those observed in Figure 5, predominate. In (**A**), the extensions of the hypertrophic astrocytes are marked in red and the microglial cells in black as a signaling guide for the structures that can be observed in (**B**) BAR: 10 microns.

As discussed with astroglia, mitochondrial dysfunction is one of the most important mechanisms for senile involution and neurodegeneration. Microglia cells may present TREM2 alterations that produce dysfunctions that induce neuro-involution [288,319,338,339]. The most striking changes in the fine and distal processes of the astrocytes and microglial cells are only observed by electron microscopy in senile animals. Figure 6 shows the increase in thickness, never seen in juvenile or adult specimens, of the finest and distal cellular processes surrounding axons, dendrites, and synaptic structures.

The joint response of all the subtypes/phenotypes of astroglial, oligodendroglial, and microglial cells leads to the maintenance of a healthy aged brain or, on the contrary, the development or acceleration of the pathogenic course of CNS diseases. New lines of research will provide new perspectives on the basic concepts of brain aging.

#### 3.2.3. Neuroglia in Human AD and AD Models

The main neuropathological characteristics of the brain in AD are the appearance of extraneuronal amyloid deposits (plaques of different types and diffuse/microgranular amyloid) and intraneuronal deposits of other insoluble and non-degradable proteins (hyperphosphorylated tau protein, alpha-synuclein) that form accumulations of different sizes (from large “neurofibrillar tangles” to small deposits that distort dendrites and axons—“dystrophic neurites”). Hundreds of thousands of works have been devoted to these deposits (origin, characteristics, and pathogenetic significance) to explain the disease and enable its therapy. However, as mentioned above, not only have the neuronal changes that lead to these neuropathological manifestations been studied but also the neuroglial changes have been studied from different points of view (from mere repercussions or collateral damage of neuronal degeneration to being the basis of the failure in neuroprotection or the direct cause of neurodegeneration). The importance given to neuroglia in AD has varied over more than a century, but it is always indisputably accepted as the major relevance of neuroinflammation in AD [339,340,341,342]. The neuroinflammatory molecules released at a significant level by astroglial and microglial cells are of prime importance in both the origin and the development of the disease. Moreover, they contribute to the modulation of characteristics of differential and personalized clinical courses in which the severity of the neuropathological changes is expressed. AD phenotypic diversity can be emphasized [343]. The amyloid deposits and neuronal dystrophies are closely related to neuroinflammatory reactions of astroglial and microglial cells. Amyloid and phospho-tau production, processing, and degradation (as well as propagation in a prion-like pathogenic disease) are closely related to the behavior and response of the neuroglial cells [253,344,345]. However, many controversies exist because we do not have a complete understanding of the different toxic or neuroprotective neuroglial mechanisms that actually occur at every moment of the disease course in every region of the affected brains. No clear differentiation between AD neuroglial alterations and the “gliosis” observed in “normal” aging has been described. This also occurs if we want to compare AD with other neurodegenerative diseases. The primary or secondary role of neuroglial responses is also discussed by different authors in the “cascades” or events that drive neurodegeneration and dementia.

In recent years, transcriptomic studies are increasingly aiming to better understand the specific changes in neurons and neuroglial cells in AD [346,347,348,349,350]. The results obtained will not only increase our knowledge about the disease but may also point to new target pathways.

The distinctive features of neuroglial cells in human AD are discussed in the following sections. Special attention is paid to neuroglial reactions in this disease not only because of its importance but because it is considered the paradigm of neurodegenerative diseases, and in studies of brain involution or degeneration, comparison is always sought with the changes observed in this pathology.

##### Astroglia

**Figure 7:**
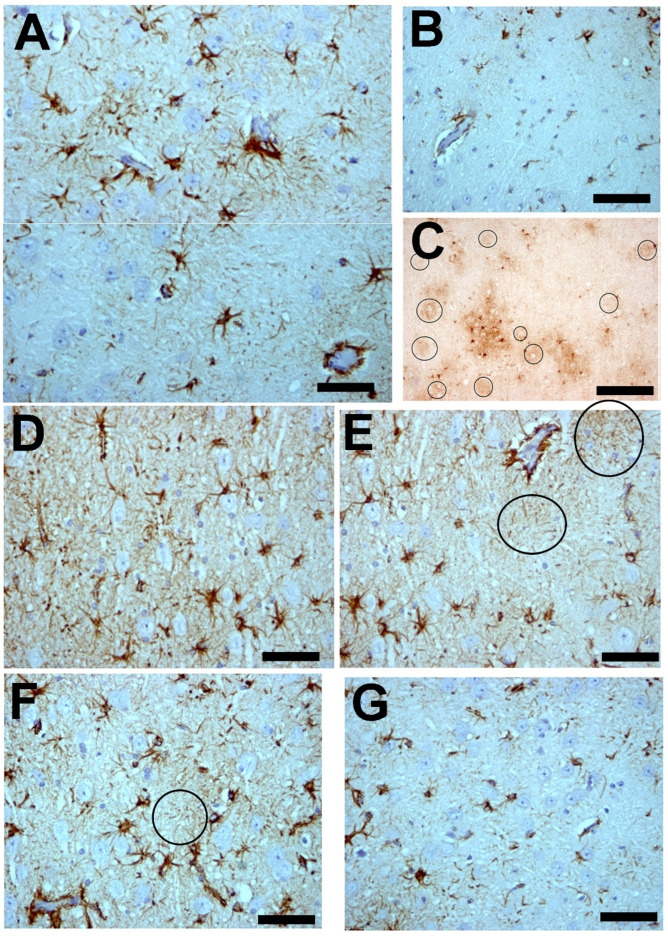
**Astroglia in Alzheimer’s disease.** Cerebral cortex, layer III, Brodmann area 46. (**A**) AD case (12 years since diagnosis) (Braak V) with subareas with high (upper area) and low (lower area) presence of reactive astrocytes. In this last subarea, the hyperreactivity of the astrocytic feet surrounding a vessel is striking. (**B**) Another area of the same case indicates truly low number of astrocytes. (**C**) Very advanced case (16 years) with little reactive astrocytes, forming clusters located randomly and without any relation to the large presence of amyloid plaques (circles) assessed in parallel sections with amyloid antibodies. (**D**–**G**) AD Braak IV case (11 years) with different degrees of astrogliosis and amyloid pathology: (**D**) area without amyloid pathology; (**E**) area with amyloid pathology unrelated to astrocytes; (**F**) area with less astrogliosis, mainly confined to forming a crown surrounding a large amyloid plaque and blood vessels; (**G**) area without amyloid pathology and little astrogliosis. GFAP immunostaining and hematoxylin contrast (except (**C**)). BAR (microns): (**A**,**B**,**D**–**G**) = 50; (**C**) = 150.

Although there are many works on the possible existence of AD-specific astrocytic reactions, there is no consensus on the subject. Authors constantly describe the existence of marked astrogliosis (“dramatic” and “generalized”) in AD [205,351]. Astrocytes undergo subtype-specific transcriptional changes in AD [349], and studies on this item will provide a more complete understanding of AD. Many regions of AD brains present a variable density of hypertrophic/GFAP-hyperimmunoreactive astrocytes (Figure 7). 

The expression of GFAP isoforms in AD cases has been described as abnormal [205,352]. In most morphological–immunoreactive studies, astroglial reactivity has been described to be strongly associated with neuropathological AD lesions, with more relevance in specific areas of amyloidogenesis in some specific CNS regions or subregions. However, all proposals have been widely debated without reaching a consensus. A1 and A2 subtypes of astrocytes have been described in some human studies [38,353,354,355,356,357]. Escartin et al. [211] consider that there are a significant number of functional subtypes of astrocytes that respond to different phenotypes. The authors suggest that when studying different astrocyte types present in a given physiological or pathological situation, they should be characterized by genetic and/or molecular markers expressed using modern techniques of analysis of isolated cells and transcriptomics and also identifying the brain region/area. This will require a new range of research but will provide an important benefit to the knowledge of diseases and their therapy. Despite all the “shortcomings” (in the face of new research with new innovative technologies), research in recent decades has provided important knowledge of astroglial responses in AD.

The most important aspects studied in AD astrogliosis are the increased number of astrocytes, special patterns in CNS regions/subregions, special association with altered neurons and blood vessels or extracellular pathologic deposits, and phenotypic changes.

Regarding the number of cells, it must be said that astrogliosis is not synonymous with hyperplasia. In most cases, what is striking is the hypertrophy/hyperreactivity of a variable number of elements (in their cell body, their cellular processes, or at their endings on the vessels) (Figure 2 and Figure 3). In various studies, it has been suggested that the number of astroglial cells in many regions of the CNS remains almost constant throughout life and, surprisingly, irrespective of the evolution of pathological disorders, including AD [358,359,360,361]. Phenotypic changes but not proliferation have been proposed as the basis of the neuroglial responses to AD [361]. In a study conducted by us on the brains of 65- and 85-year-old AD patients (with a disease course of 10-15 years) in parallel to age-matched healthy controls, we observed that the number of GFAP-positive astrocytes in AD (65 and 85 years old)-affected brains was less pronounced than that observed in normal 85-year-old individuals [204].

The special patterns of astrogliosis described in some CNS regions of AD brains, as mentioned above, have been discussed in other studies. The proposal that cortical astrogliosis in AD follows a laminar pattern, with two reactive bands in layers I-III and in layer V [253], has not been accepted by other authors. This pattern helps differentiate AD and normal aging (which is often associated with at-random cortical gliosis [362,363]). Subcortical gray matter astrogliosis is commonly observed in both normal aging and AD. In our studies, the named “high variable” and "disorganized” astrogliosis is more often observed in AD than in aging brains [204], even in areas where astroglial cells are lost to a large extent [204] by different mechanisms (senescence, atrophy, and asthenia; [207,364,365,366]). Hypertrophy is more pronounced in the white matter (where there is a greater number of fibrous astrocytes), as well as at the interface between the gray and white matter. Several authors consider that many areas lacking reactive astrocytes and with low reactivity of normal astrocytes are zones of accelerated neurodegeneration, in which senescence, atrophy, or dystrophy of these astroglial cells occurs [7,253]. In them, neuronal pathologies can originate or activate since the maintenance of normal astrocytes is necessary for the normal activity of neurons. The adaptive changes of neurons are largely induced by neuroglial cells. In AD brains, astrogliosis is quite variable when different brain regions are studied. Probably, it reflects the state of involution in each brain and/or in each region of each brain induced by local pathological features. However, we have observed that the areas of greater or lesser degree of astrogliosis in different regions of the cerebral cortex, hippocampus, and cerebellar cortex alternate randomly when large regions/areas are studied in serial histological sections. A complex age-dependent remodeling of these cells in different areas of brain regions/subregions should be produced. All this suggests that astrogliosis is not a phenomenon that occurs homogeneously and that there are foci of increased risk or less degeneration in each region of the CNS. Likewise, it suggests that astrocytic responses can be heterogeneous within a region/subregion of the CNS (or that they can have a marked “personal” accent in different elements or subgroups). A more or less generalized/localized neurotoxic or neuroprotective framework can induce different patterns.

Reactive astrocytes are described as close to both different types of β-amyloid plaques and tangle-bearing neurons. The cortex and hippocampus have been the CNS regions better studied (Figure 2 and Figure 3). The plaque and astroglial cell relationships are complex and variable. Very different descriptions can be obtained in the bibliography (more in the same studied brains): plaques with a scar-like crown of astroglia, plaques with at-random isolated peripheral astrocytes, plaques infiltrated by astroglial processes, plaques not associated with astroglia, etc. The significance of reactive astrogliosis around the plaques is unclear, and different authors claim a noxious or protective interpretation [19,211,359,367]. A weaker astrocyte response against amyloid deposits has been associated with cognitive impairment, and this “loss of defense response to neurodegeneration” has been considered a main component of the disease [368,369,370]. In a contrary sense, high reactive astrogliosis in the cortical and/or hippocampal-affected areas can be considered to be harmful. However, this astrogliosis can be beneficial if it tries to normalize the tissue. One study concluded that the greatest neurotoxic plaques are those that have reduced contact with astrocytes [370,371]. Morphological associations between neurons with tangles and astroglial cells have been described [19,112,371] (Figure 2), and a clear pathophysiological functional interdependence between such neurons and reactive astrocytes has been proposed [10,111,112,359,371]. 

The most important and defining changes in diverse functional subclasses of astrocytes in AD are yet to be discovered. Phenotypic changes will lead to selected neurotoxic or neuroprotective responses. Complex and region-specific changes of specific markers of A1 and A2 astrocytes have been described in both the aging brain and AD [206,372], presenting a general prospect of pathogenic changes. Astrocytes developing pathological phenotypes could contribute to AD progression [373]. However, the exact astroglial responses and brain effects will be further specified with new analyses of gene expression, transcriptomics, and molecular secretions. The effects may be local, acting on neurons or neuroglial cells in the environment, or they may be more general, acting at greater distances and over a longer term with the emission of vesicles and exosomes carrying neuroprotective or neurotoxic, pro-inflammatory or anti-inflammatory, and pro-oxidant or antioxidant substances. Extracellular astrocyte vesicles from human AD patients and AD models (3xTgAD mouse) have been shown to transport degenerative substances for neurons, glial, and endothelial cells, aggravating AD. Zones with a high density of immunopositive-GFAP reactive astrocytes are observed to not be associated with dystrophic neurons or amyloid plaques. We do not know the phenotype or their involvement in the pathogenic or neuroprotective mechanisms of these cellular elements.

Aberrant reactive astrocytes can be seen in AD human brains. Several dispersed or grouped astrocytes accumulate amyloid or aberrant tau protein deposits, similar to what was observed in experimental AD models and in animals that present AD-like amyloidosis (especially monkeys and simians) [374,375,376]. Astroglia may generate amyloids; in this way, these cells can participate in the generation of amyloid that leads to AD. Hypertrophic astrocytes with abnormal gene expression, such as calretinin, have also been described by us [204]. These diverse astroglial responses observed in AD patients and post mortem AD brains could be a reflection of the multifactorial nature of this disease [19,367].

Finally, although of primary importance, we would like to point out that astrocytes have capital importance in the formation and maintenance of the blood–brain barrier (BBB) (mentioned above in several sections) and the so-called neurovascular units. Both morphological and functional alterations have been reported in AD. Alterations in the thickness of the endothelium, the basal membrane, and the perivascular astroglial terminals (end-feet) are manifested (Figure 2 and Figure 3).

##### Oligodendroglia

Although demyelination is a secondary characteristic feature of AD, alterations to oligodendrocytes have rarely been studied in this disease. For in vivo AD diagnosis, demyelination assessment using brain-imaging techniques is widely used. An association of amyloid pathology with myelin alteration in human preclinical and diagnosed AD, as well as in AD models, has been confirmed [125,377,378,379,380,381], and remyelination could be a therapeutic strategy for AD 124]. Metabolic changes in AD oligodendrocytes have been observed, such as alterations in glycolytic and ketolytic gene expression [185]. Oligodendrocytes undergo subtype-specific transcriptional changes in Alzheimer’s disease [349].

The decreased regenerative capacity of NG2-Glia/oligodendrocyte precursors in AD subjects, as well as in AD models, has been pointed out as a main cause of the neurodegenerative processes [379,382,383,384,385,386]. Senescent OPCs can be produced. However, the significance of these alterations (neuroprotective or neurodegenerative) is not completely clear. Oxidative stress and inflammatory tissular scenarios can be conducive to the occurrence of cytotoxic involutional alterations in OPCs [385]. The disruption of oligodendrocyte progenitor cells is an early sign of pathology in the triple transgenic mouse model of Alzheimer’s disease [385]. Senolytic therapy alleviates Aβ-associated oligodendrocyte progenitor cell senescence and cognitive deficits in an Alzheimer’s disease model [240].

##### Microglia

In many studies, neuroinflammation is considered one of the main mechanisms driving the pathogenesis of AD [19,339,340,342,367,387,388,389], and microgliosis is the main manifestation of the pathogenic machinery put into play to lead to AD. However, these processes can also explain degenerative changes in aging and in other neurodegenerative pathologies [19,367], and furthermore, in a contrary functional sense, they could protect against neuronal degenerative changes (via phagocytosis of damaged neurons or amyloid plaques [390] or via the production of neuroprotective agents [391]. Microglia work to reestablish normality and to stop further degeneration in the initial stages of AD but mainly fail in the illness’s later phases [392]. Reactive microglia provoke neurodegeneration through an excess of neurotoxins released in the neural microenvironment (chemokines, cytokines, or inducers of oxidative stress) [387,388,389,392]. 

AD is considered to have a high degree of microgliosis, higher than that observed in normal aging and in some other neurodegenerative diseases. But this widespread belief has been disputed in important aspects, as we discuss in various paragraphs. The microglial response varies during the onset and course of the disease. Microglia initially try to counteract degeneration until they fail, and their toxic responses contribute to aggravating the disease. Finally, there is "aging", "exhaustion", and microglial death, with a characteristic decrease in these cells in neuropathological studies. The sequence of appearance of the microglial reaction and that of the disappearance of normal microglia in human AD has been discussed in assorted studies [85]. There exists a high turnover of microglia in the human brain [393]. On the other hand, it should be noted that, as described above, microglial cells can have many physiological and pathological functions: the regulation of synapses and their function; the regulation of homeostasis, phagocytosis, neurorepair, and neurotoxicity; the triggering of neuroinflammation; and the promotion of anti-inflammation. Several morphologies are more suggestive of some of these functions (rounded shapes with no or few extensions are more typical of phagocytic cells, and cells with large, highly branched extensions seem to be pro-inflammatory), but this cannot be confirmed until their phenotype is known [394].

Many authors consider a significant increase in the number of both resident and reactive microglial cells in the parenchyma, as well as around the vessels or in association with neuropathological lesions, in AD brains. The specific accumulation of microglial cells was described to be strongly associated with amyloid deposits, even infiltrating amyloid plaques [395,396]. This association is interpreted in two ways. On the one hand, microglial activation in AD seems to be Aβ-dependent, with Aβ binding to scavenger receptors [393] and Toll-like receptors (TLR2, TLR4, and TLR6) [397], as was demonstrated in mouse models of AD. On the other hand, the phagocytic defensive function of microglia would mobilize microglial elements toward the areas of amyloid deposition to eliminate these pathological accumulations [387,398]. Plaques with high accompaniments, with little accompaniment, or with no accompaniment of microglial cells (rounded and/or elongated-branched shapes) actually coexist with no convincing explanation. Neuropathological studies show us a frozen snapshot of a dynamic process subject to variable local factors, and the statistical analysis of a considerable number of pathological structures shows us the tendency of the pathological course of the studied region. 

**Figure 8:**
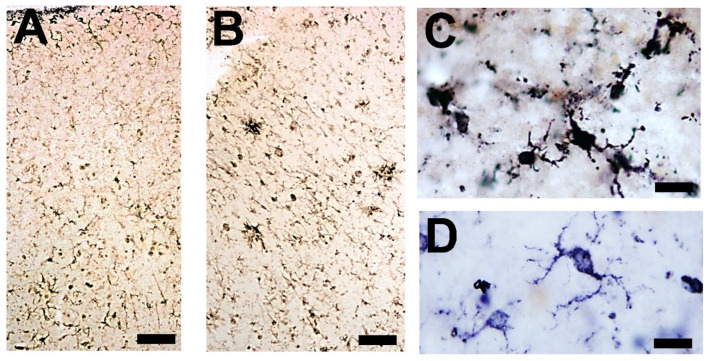
**Microglial cells in Alzheimer’s disease**. Cerebral cortex, layers II-IV, Brodmann area 46. (**A**) AD case (12 years since diagnosis) (Braak V). (**A**) Microgliosis in a wide area without amyloid plaques. (**B**) Microgliosis in an area with amyloid plaques; many plaques are infiltrated by microglia, and many cells are more intensely inmmunostained. (**B**) Different subtypes of microglial cells are highly intermeshed and located at random. (**C**,**D**) Highly hypertrophic/hyperreactive cells of different morphologies in two different areas of microglial density. Iba-1 inmunostainning. BAR (microns): (**A**,**B**) = 200; (**C**,**D**) = 20.

Patterns of microgliosis are not generally accepted in AD. However, a higher density of microglial cells has been described in several areas, such as subpial zones or the transition between the neuronal and molecular layers of the hippocampus and cerebellum [204]. In serial section neuropathological studies of the brain, the reactive microglial cells can be observed to be variably accumulated in more or less severely affected areas. More reactive cells can be observed in areas with more neuropathological expression (Figure 8). In some models of AD, microgliosis is observed before the onset of AD pathology. This fact suggests that inflammation/microgliosis is in the origin of AD. Significantly, beta-amyloid immunotherapy seems to down-regulate microglial activation and reduce the inflammation induction of AD [390].

Different morphological and/or phenotypical reactive microglia have been described in different regions or subregions of the AD brain [399]. Brains affected by AD display different morphological subtypes of microglial cells in different regions (cortex and hippocampus) (Figure 3 and Figure 8), and hypertrophic and highly ramified extensions scattered throughout the parenchyma have been observed, in addition to cells with poorly branched processes and cells with a round morphology. The forms with prominent extensions can also invade several subtypes of amyloid plaques or are closely related to blood vessels or dystrophic neurons. The round forms are mainly located close to the plaques or blood vessels, but we can also observe normal or hypertrophic elements of this type diffusely distributed in the brain parenchyma (Figure 3 and Figure 8). 

In a recent study conducted on subjects with advanced AD, a decrease in ramified microglia (considered a characteristic of normal/healthy brains) was described [243]. The authors considered that activated microglia were not associated with AD; the reactive elements were only increased in nondemented controls but with a stronger AD-type pathology. Moreover, the authors considered that microglial reactive clusters were only occasionally associated with plaques (composed of Aβ and tau-positive dystrophic neurites) in less than 2% of the total microglial population. In our studies, we have shown that the number of microglial cells in the cerebellum and hippocampus is more closely related to the age of the individuals than to their AD pathology [204].

Very different microglial phenotypes have been described in AD brains, but no clear microglial responses have been associated with specific alterations, specific areas of the brain, or specific phases of the progression of AD. However, this issue is now being widely studied because the greater knowledge of the disease and its different clinical subtypes, as well as the personalized therapy for each of them, depends on this detailed study. Using single-cell RNA sequencing, a subset of microglial cells enriched for AD disease-related genes and RNA signatures has been described [400,401]. The genotype of microglial cells in different individuals is considered closely related to AD progression [402]. This has been confirmed in AD models. Microglial TREM2 facilitates the adaptive regulation of amyloid plaque formation and reduces the local induction of neurodegeneration. However, some TREM2 variants, such as the R47H variant, are associated with a higher risk of AD [403,404,405,406,407,408,409]. Increased AD risk is induced by other polymorphisms in genes exclusively or largely expressed in microglia, such as CD33, CR1, ABCA7, and SHIP [401]. Recent works report that human and mouse single-nucleus transcriptomics differences in two types of cellular responses in AD: TREMP2-dependent and TREMP2-independent [408]. Moreover, TREMP2 haplodeficiency impairs microglial barrier function, decreasing amyloid compaction [410]. 

“Aged microglia” not only affects synaptic function [7,20,121,285,334,335,336,337,338] but have demonstrated that neurodegeneration is secondary to aging-related microglial involution [207,359]. There exists a programmed death of microglia in AD [7,288,334,335,336,337,338,400]. The inability of microglia to remove amyloid deposits has been considered a cause of “microglial exhaustion”, which in turn promotes “neurofibrillar neurodegeneration, brain failure and dementia” [207,334,335,336,337,338,359].

The alterations in the microglial cells strongly alter the glia crosstalk which is a main promoter of neuroinflammatory diseases, AD in special [411].

There are numerous AD models that have been extremely useful both in verifying and deciphering the complex physiological and pathological mechanisms of glial cells and discovering new pathogenic pathways in which these cells are involved. There are animal models produced by neurotoxins, surgical interventions, trauma, and so on. Undisputedly, genetically modified animals are the most commonly used today: silencing or elimination of genes or, on the contrary, the insertion of human genes involved in AD pathology. The results of research with these models have provided advanced knowledge about AD and also make possible new avenues of treatment. Innovative technologies (single-cell analysis, gene expression analysis, transcriptomics, proteomics, etc.) will greatly expand the impact of studies with these models by better defining the responses of neuroglial cells, characterizing subtypes, and correlating local and regional pathology with glial changes.

**Figure 9:**
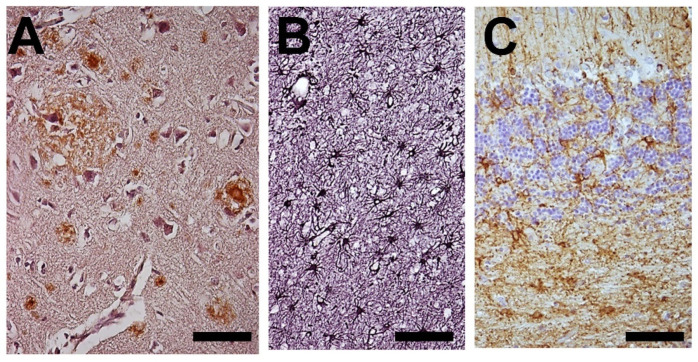
**Neuroglial reactivity in the hippocampus and cerebellum.** (**A**) Hippocampal CA3 region in a case of Braak IV human AD. Amyloid plaques of very different types are not accompanied by neuroglial cells; neuroglial nuclei are not evident (Bielchowsky’s silver-impregnation technique). (**B**) Hippocampal polymorphic sub-CA3 region of another case of Braak V. A large, very dense collection of small reactive astrocytes is observed. There are no amyloid plaques, and very few neuronal profiles are seen. (**C**) Cerebellum of a Braak V case of human AD. Hypertrophic hyperreactive astrocytes (including Bergman fibers) in the three layers cerebellum and in the white matter. There is no amyloid neuropathology. In the Purkinje layer, nuclei of immunonegative GFAP astrocytic cells are seen (astrocyte hyperplasia). BAR (microns): (**A**) = 200; (**B**) = 75; (**C**) = 50.

The vast majority of studies on the neuro- and gliopathology of Alzheimer’s disease in humans and experimental models have been conducted on the frontal cortex of the brain. The hippocampus and temporal cortex of the brain have also been studied but to a lesser extent. Other regions, such as the cerebellum, which do not usually present classic neuropathological alterations, have been studied to a lesser extent. Studies on the cerebral cortex, applied statistically, show a general panorama of neuroglial responses that are commonly accepted, but one should not forget, as we have pointed out in this review, the large local and regional variations in the astroglial reaction. Certainly, these variations must have a neuropathological significance. Astroglial and microglial reactions in the hippocampus are usually considered to be always constant and similar to those observed in the cerebral cortex and manifest themselves in all its different regions. But this subject is not fully studied and is highly debatable. In many cases (Figure 9), areas with high neuropathological alteration show a high decrease in neuroglia. On the other hand, the large hypertrophic/GFAP hyperreactive cells are observed to be very dispersed and isolated, most of them related to plaques, but without being considered in the existence of generalized astrogliosis [7,19,20]. In selected cases, minority, large groups of reactive astrocytes (Figure 9) have been shown in some regions of the hippocampus (similar to those observed in experimental models—Figure 2), which are difficult to interpret. In the cerebellum, lacking amyloid and phospho-tau-dependent neuropathological alterations, significant astroglial and microglial reactivity has been observed [207] (Figure 9). Both the different relationship between neuropathological manifestations and local neuroglial reactions in the brain cortex and hippocampus and hyperreactivity in the cerebellum, which does not present important neuropathological manifestations, may suggest that there are inducers of AD neuropathology at a higher level than the neuroinflammation caused by neuroglia (genetic/epigenetic; T2D and metabolic syndromes, mitochondrial dysfunctions, etc.) that will ultimately be the most important tool or mechanism to reach the ceiling of brain neurodegeneration (AD), even employing long-distance dissemination of promoters of neuronal involution and neuroglial reactivity. The CNS is not a homogeneous system and involutes or undergoes specific pathological changes in different regions and in different situations.

#### 3.2.4. Neuroglia in Human Obesity and Diabetes and Obesity/Diabetes Models

**Figure 10:**
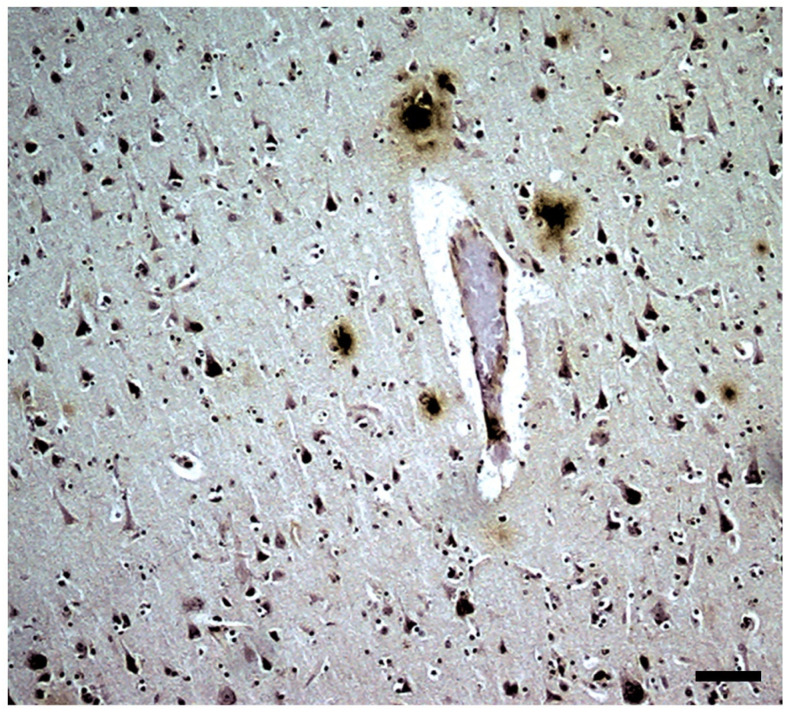
**Amyloid plaques in type 2 diabetes**. Cerebral cortex of a T2D case (12 years of disease course and 3 years of cognitive impairment). Amyloid plaques (stained with amyloid antibody 6E10) are seen, but no infarcts are evident. Blood vessels appear dilated or surrounded by empty spaces. BAR: 80 microns.

In the subchapter dedicated to common T2D and AD pathogenic mechanisms, the main pathogenic features of T2D are presented. In a neuropathological sense, even considering the peculiarities of each of the studies (or those of some of the cases analyzed in certain studies), the observed human postmortem pathology in T2D individuals is between that observed in the senile brain and those that manifest in AD and/or vascular dementia (VD). The descriptions are focused on amyloid plaques, vascular amyloidosis, neurofibrillary tangles, and perivascular and BBB alterations. All of these alterations coincide, in a first approach, with those observed and described in AD. Regarding the similarity with AD, it is especially important to note that there are neuronal alterations similar to those described in AD in cases when T2D cases with low or no cognitive and neuropathological alteration (Braak 0-II) [33]. In general, there is a lower manifestation of neuronal changes and deposits of fibrillar proteins when compared to AD (Figure 10).

Some authors consider that there has been an “advance” in the death of the case study due to the metabolic disease. Regarding differences with AD, two aspects are worth highlighting: (a) amylin deposits and (b) important neuropathological signs of cerebrovascular dementia.

(a) Although it is not a differential characteristic of T2D, a very marked immunoreactivity against the amylin peptide is observed in most of the brain cases of T2D [412]. This characteristic is increased in T2D-AD cases [412,413,414,415], perhaps due to genetic reasons. Amylin (islet amyloid polypeptide; IAPP) is a pancreatic β-cell hormone, co-secreted with insulin, which regulates glucose homeostasis [416]. However, in individuals with pre-diabetic or diabetic insulin resistance and hyperinsulinemia, amylin is oversecreted and tends to be deposited in various tissues (only in humans, not in rodents, unless a human gene has been introduced into the latter), with special repercussions in the brain (where it enters by crossing the BBB). Amylin dyshomeostasis can be a possible new link between T2D and an increased risk of AD [412]. This deposition of pancreatic amylin is added to beta-amyloid deposits. The deposited amylin produces neuronal degeneration, apoptosis, and neuroinflammation and interacts with the metabolism of the tau protein and its phosphorylation [413]. Amylin and amyloid β interact at the molecular level, increasing deleterious effects [417]. There is also cerebral production of amylin of neuronal origin, which would explain the immunoreactivity in brain neurons, although it is not considered very pathogenically important in certain studies [415]. Amylin can be a potential link between type 2 diabetes and Alzheimer’s disease [412,418,419].

(b) Closely linked to hyperamylinemia and the appearance of amylin deposits in the brain, according to several authors [419], signs of microvascular lesions and alterations typical of cerebrovascular pathology are observed in many cases of T2D (especially lacunes [33,420,421,422,423]). Amylin alters the vascular network in the brain [419,424]. As discussed above, in certain studies, it has been considered that brain damage caused by metabolic diseases (including T2D) leads to vascular dementia and not AD. It has also been speculated that there may be accelerated neurodegeneration caused by T2D via non-AD mechanisms [33].

The literature on neuroglial changes in human T2D brains is not very abundant, but important works on alterations in astroglia can be mentioned. Especially relevant is the aforementioned by Bury et al., 2021 [115], which shows alterations in the gene expression of genes in astrocytes closely related to blood vessels and in endothelial cells of these vessels. Alterations have also been described in oligodendroglia cells. But little is reported in human studies on alterations of microglia in nervous tissue. However, astroglial and microglial alterations (or, rather, the toxic responses of subtypes or populations of these cells) are of capital importance in the genesis and development of metabolic pathologies (obesity, metabolic syndrome, and T2D). Cytokines and glycated end products originating from inflamed peripheral tissues cause alterations in the BBB and brain nervous tissue (in neurons and neuroglia cells). To this initial induced (external) neuroinflammatory process, the internal neuroinflammatory process of the brain will be added, in which astrocytes and microglial cells play a leading role. On the other hand, the alterations induced by insulin resistance and hyperglycemia, typical of T2D, must have a logical response in the glial cells. But there are no studies on this aspect. It should be noted that various studies have pointed out important differences between the sexes in T2D and its transition to T2D-AD [425].

Studies on obesity and T2D models have provided diverse theories for brain degeneration in metabolic diseases. There are different models for these studies, produced especially in rodents [426,427], by high-caloric diets (sugars, fats), cytotoxic drugs, or manipulation of genes related to obesity or diabetes. Each of the models has its advantages and disadvantages. T2D models produced by cytotoxic drugs, such as Streptozotocin, always leave the doubt of whether the induced pathological changes are due to the substance used or to the obesity/diabetes caused. Most studies on obesity and T2D models do not carry out an in-depth analysis of neuroglial cells, although it is assumed that neuroinflammation is a pathogenic mechanism of primary importance in the brain pathological process. In all the studies, the reactions of astrocytes and microglia cells determine the neurodegeneration and cognitive deterioration of the models.

Limited Alzheimer-type neurodegeneration in experimental obesity and type 2 diabetes has been observed in some models [428], but important neuropathological alterations, many of them compatible with those described in AD, have been observed in the majority of the models studied in late life. Increased Aβ production prompts the onset of glucose intolerance and insulin resistance [429]. The accumulation of amylin, the combination of amyloid and/or tau deposits, brain microvascular injury, and white matter disease have been described in different models of T2D [415,417,419].

In models induced by a high-calorie diet, it is essential to point out that the dysfunctional alterations in the brain when the carbohydrate and lipid supply to the brain changes initially occur at the hypothalamic level and in the circumventricular brain regions where the permeability of the BBB is physiologically increased (see Section 2.1). In a very remarkable way, the astroglia of these sensitive hypothalamic areas undergo especially important changes/reactions that induce neuronal alterations in the neuronal nuclei with which they are associated. This gives rise to changes in the expression of hormones that regulate the energy control of the brain and other systems of the organism. If the adaptive mechanisms of the CNS cannot normalize the initial dysfunctional alterations, or if the diabetogenic inducers persist, pathogenic degeneration of the brain develops (or AD neurodegeneration and/or vascular dementia are induced). This “dissemination” of neurodiabetic pathology and/or the way in which a global AD neuroinvolution is induced in the brain is not well known, but there are absolutely conclusive studies that in shorter or longer times, there are degenerative/involutive changes in many regions of the CNS of the studied models (especially in the hippocampus and frontal cortex) that are then observed with similar characteristics in human studies. As a pre-diabetic state develops, insulin resistance develops, and hyperglycemia becomes established, and when overt T2D is established, widespread attacks on the BBB in different regions of the CNS allow an all-out offensive to the brain nervous tissue mediated by cytokines, lipids, and AGES. In these diet-induced obesity/diabetes models in normal animals and also in genetically induced models (db/db), activation and/or dysfunction of astroglial and microglial cells are present. In an obesity model, it has been seen that the cephalic phase of insulin release, impaired in obesity, is modulated by IL-1β originating from microglia. Moreover, IL-1β activated the vagus nerve to induce insulin secretion and regulate the activity of the hypothalamus in response to cephalic stimulation [430]. Phenolic substances can induce changes in insulin resistance and microglial responses [431]. Action on selected microglial receptors (such as CX3CL1R) protects from diet-induced obesity [432].

Astroglial cells suffer from insulin resistance and are sensitive to hyperglycemia. In vitro and in vivo studies show that the functions of different intracellular signaling systems in astrocytes are modified [433]. These reactive astrocytes may show some morphological changes (alterations in the radial processes—vesiculations and accumulations of gliofibrils) but, in particular, they present very relevant phenotypic changes that cannot be observed unless very sophisticated molecular or genetic-analysis techniques are used. As a result of these astroglial changes, an increased release of cytokines and chemokines, NO, oxidative/nitrosative reactive products, and other pro-inflammatory molecules is noted. It should also be considered that astrocytes have a key role in the regulation of cerebral blood flow, in the elimination of toxic elements via the glymphatic system, and in the permeabilization of substances through the BBB. All these functions are altered. High glucose impairs the astrocyte functions and the function of BBB [434]. Metformin has anti-inflammatory properties in cultured astrocytes in a medium with a high glucose concentration [435].

A review [436] has shown that oligodendrocyte precursor cell dysfunction can be responsible for neurobehavioral deficits in diabetes, and therapeutical interventions have been directed in this way.

Microglial cells seem to undergo a reactive activation, integrated into the neuroinflammatory reaction triggered by peripheral cytokines and the abnormal astroglial response. A reduction in the microglial response has been revealed around amyloid plaques but not in the affected brain tissue [437]: this observation may translate to a lower capacity of reactive microglia to phagocytose the accumulation of amyloid protein deposited in plaques.

Single-nuclei transcriptomics of the mouse cortex reveals that dietary obesity involves microglial activation and promotes transcriptional dysregulations in neurons and glial cells. The transcriptional dysregulations of microglia, more than of other cell types, are like those in AD, as assessed with single-nuclei cortical transcriptomics in a mouse model of AD and two sets of human donors with the disease [438]. Gene expression changes in astrocytes and microglia from high-fat diet obese/T2D animals are similar to those observed in human Alzheimer’s astroglia and microglia, as well as in diabetic and obese individuals before AD development [439].

Many study models, both in T2D pathology and in T2D-AD treatments, have actually been carried out in modified AD models in which metabolic/diabetic changes were induced. This will be presented in the next section.

#### 3.2.5. Neuroglia in Human AD-T2D and Models

The involvement of T2D in the induction of AD-type neuropathology has been controversial for years, as we already reflected in Section 2.2. While postmortem data from animal studies have supported the involvement of T2D in AD-type neuropathology through common mechanisms that may affect the development of neuritic/amyloid plaques and neurofibrillary tangles, findings from postmortem studies in humans have provided inconclusive data on the association of T2D with AD. To complicate matters, medications to treat T2D have been implicated in reduced AD-type neuropathology and life span. In studies on human brains, there is a very important bias when trying to reach general conclusions due to different causes: (i) the authors’ prejudices/preconceived ideas about whether cognitive decline is an Alzheimer’s-type neurodegeneration, vascular, or mixed type, or special T2D-induced dementia; (ii) whether the T2D pathology leads to the end of life when neurodegeneration has not yet been defined; (iii) whether a severe vascular dysfunction of the brain (including brain infarcts and stroke) and other systemic cardiovascular problems is the most important pathological problem; (iv) whether the observed neuropathological picture responds to the medication received to treat T2D (in both positive and negative sense) and/or the duration of T2D (including the obesity period). In the following paragraphs, we will try to clarify these issues in a discussion. In any case, in all studies, it has been observed that, to a greater or lesser degree, cognitive impairment or dementia always presented signs of AD pathology. We must also highlight that in human studies, little attention has been paid to neuroglial changes or reactions, although in the analysis and discussion of results, the importance of neuroglial reactions and neuroinflammation is always taken for granted.

First of all, we can indicate that the incidence of medication, or non-medication, of T2D and the duration of the disease have been considered of prime importance in human studies on T2D-AD. However, few studies have examined relationships between diabetes medications and neuropathological outcomes in humans. These efforts have been constrained by difficulties in adjusting for diabetes severity and duration. In a study published in 2021 [440], no significant association was found between any drug class and traditional neuropathologic AD presentation (Braak V–VI) compared to nonusers of that class. However, five years of sulfonylurea and biguanides use was associated with lower levels of Aβ1–42 compared to nonusers, which was not the case with patients treated with insulin. The decrease in Aβ1–42 levels should correspond to a decrease in neuropathology, but this is not constated in the study. The authors consider that there is consistent evidence showing a connection between medicated/nonmedicated T2D and cerebral infarcts but not AD neuropathologic change. This supports a role for cerebrovascular disease in relating T2D to “ADRD” (Alzheimer’s Disease and Related Dementias). This study considers the existence of advanced AD neuropathology (although it does not analyze neuroglial changes) but focuses on the possible prevention of cerebrovascular disease via antidiabetic medication. Previous works from 2008 [441] and 2014 [420] considered that there was a significant relationship between T2D and cerebrovascular disease (dependent on antidiabetic medication) and that the implication in the appearance of AD neuropathology was questionable. In the study, they found that the brains of T2D patients who were treated with both insulin and a hypoglycemic medication had 80% lower neuritic plaques in the hippocampus and several cortical regions compared to non-T2D and subjects who received either insulin or a hypoglycemic medication.

In a 2009 study [104] comparing the brains of patients with and without T2D, it was concluded that T2D was more related to cerebrovascular dementia than to AD. This was apparently confirmed by other studies [442]. However, the data provided show a fairly similar AD-like neuropathological picture in both groups. CERAD and NIRI scores were significantly higher in non-diabetics, but the Braak grade was similar (II–III), as were NFT and plaque counts in several brain regions. In any case, it has long been considered that mild cerebrovascular dysfunction can lead to the development of Alzheimer’s disease. Only in selected papers related to human and T2D-AD models was the role of glial cells considered, such as in a study in which the diabetic phenotype in mouse and humans reduced the number of microglia around β-amyloid plaques [437] or in other studies related to the responses in astroglial cells [210,443,444].

An important critical review by Biessel and Despa in 2018 [33] on the topic of the T2D-AD relationship includes these literal considerations: “Converging evidence from brain autopsy studies from the past decade shows that the core neuropathological features of AD are not more common in subjects with than in those without T2DM (T2D in this review)” but “over 40% of individuals with T2DM have intermediate to severe AD pathology in their brain at the time of death. Yet, the elevated dementia risk in T2DM should be attributed to pathologies other than AD, which may often evolve on a background of AD pathology. This clearly includes vascular disease, but also non-AD mechanism of neurodegeneration”. This paper also mentions the results of studies in animal models, clearly indicating the close relationship between T2D and AD, the involvement of neuroglial cells, and the alteration of the blood–brain barrier in T2D-AD association.

As mentioned above, the deposition of pancreatic amylin is added to beta-amyloid deposits, increasing the amyloid burden. The deposited amylin increases neuronal degeneration, apoptosis, and neuroinflammation, as well as interacts with the metabolism of the tau protein and its phosphorylation [413]. Amylin and amyloid β interact at the molecular level, increasing their deleterious effects [417].

It can be concluded that neuropathological studies in humans who have suffered from T2D show the existence of alterations suggestive of AD in most cases, although in variable proportion, together with cerebrovascular changes. Most studies have been devoted to analyzing, using diverse types of neuroimaging (especially magnetic resonance imaging), alterations in gray and white matter and strokes in various brain regions. In the neuropathological field, a few morpho-histochemical studies on T2D brains have focused on the study of the existence of infarcts and perivascular lesions as well as the existence or not of amyloid plaques or neurofibrillary tangles. It can be surprising that there are no studies dedicated to highlighting changes in neuroglial cells, not even as accompanying responses to neuronal degeneration. However, what is incontrovertible is that, in all cases, neuroglial cell involvement is totally accepted. This is attested in a plethora of studies and reviews on the causes and mechanisms of action of the neurodegeneration that underlies T2D and T2D-AD brains, but these works, to a significant extent, are based on research carried out on animal models (rodents—[71,389,433,443,444,445,446,447,448]; monkeys—[449,450]. These statements are all applicable to the BBB changes [451].

In a similar way, the most reliable data on the relationships between these two pathologies (AD and T2D) and the existence of common pathogenic mechanisms in them will be obtained from studies of these double models since, as has been shown, there is a great deal of conflict in the analysis of results when studying human brains.

Most of the studied T2D-AD models have aimed to increase knowledge in two aspects of foremost importance in the study of T2D and AD relationships. (A) Firstly, an attempt has been made to clarify the relationships between these two pathologies, investigating whether there is a marked increase/acceleration of AD neurodegeneration in AD models in which a diabetic pathology is induced by various methods (especially by hypercaloric/diabetogenic diets) [32,422]. (B) Secondly, studies have been conducted to verify whether antidiabetic treatments can be beneficial to treating AD or the combination of T2D-AD pathologies.

(A) In the first aspect mentioned, most of the works have shown an unquestionable relationship in pathological concurrence of T2D and AD. This relationship has been shown in different pathogenic mechanisms and with variations and nuances in the different models studied. Likewise, cross-influences between the two pathologies have been described. In a work by Velazquez et al., 2017 [452] carried out on transgenic mouse models (Tg2576 and 3xTg-AD mice), the existence of insulin signaling and energy metabolism abnormalities in the brain months before peripheral insulin resistance was shown. The authors point out that there are different signaling networks that influence CNS metabolism, which, in turn, may alter peripheral insulin signaling. Early alteration of energy metabolism in the hippocampus of the APP/PS1 mouse model of AD was described [453]. An important number of studies have shown a clear increase in AD neuropathology (amyloid deposits and tau pathology, as well as alterations of the brain vasculature) when diabetogenic interventions are conducted [32,439,454]. A high-fat diet increases amylin accumulation in the hippocampus and accelerates brain aging in hIAPP transgenic mice [455]. Sometimes, the results are contradictory: in a study on 3xTgAD mice, a high-fat diet induced memory impairment but without changes in amyloid and tau pathology [456], but another study on the same model found an increase in amyloid and phospho-tau neuropathology [457]. Prediabetes-induced vascular alterations exacerbate neuropathology in APP/PS1 mice [106]. In a transgenic mouse model of AD (Tg2576), a high-fat diet that has proven to induce insulin resistance has been able to stimulate the generation of amyloidogenic Aβ peptide, activate GSK-3α and GSK-3β associated with γ-secretase activity, and decrease IDE activity (Ho, 2004). In this model, the reduction in IR decreases the production of neuropathology [458].

In an animal model presenting both AD and T2D (by crossing APP/PS1 mice with db/db mice), in which T2D debuted at 4 weeks of age and AD (amyloid plaques) at 14 weeks of age, significant brain atrophy and tau pathology were detected in the cortex at 14 weeks and the hippocampus at 26 weeks, together with an increase in microglial activation. Moreover, it is suggested that blood–brain barrier alterations may be responsible for the early pathological features observed, and the change in metabolic parameters can predict many of these alterations. Exacerbated pathology and hemorrhagic burden were present in this T2D-AD model [106].

Many authors consider that the best animal models of T2D-AD are high-fat/caloric-diet-AD models [32,439]. The studies conducted in this field actually compare the AD model (which receives a control diet) with the double T2D-AD model (which receives a diabetogenic diet). Consequently, they will provide us with essential information about AD and T2D-AD.

**Figure 11:**
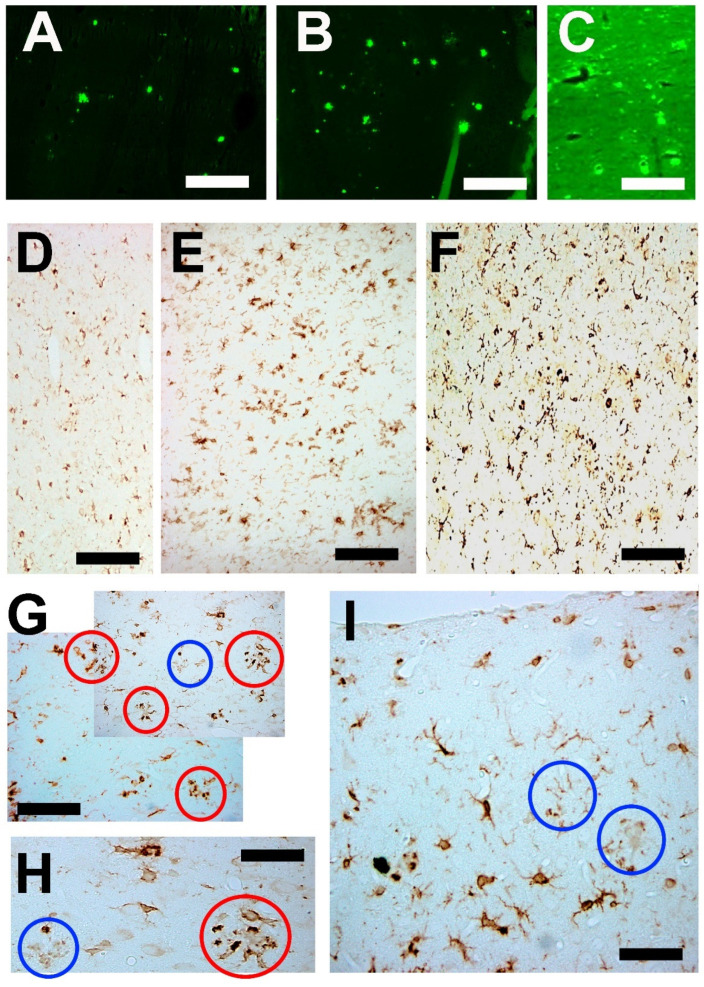
Effect of long-term hypercaloric diet on brain neuro-gliopathology in an experimental Alzheimer’s model (APP-PS1, double-transgenic mice). (**A**,**B**) Amyloid plaques and smaller granular deposits at 6 months of age in layers IV-V of the frontoparietal cortex (**A**) normal diet; (**B**) hypercaloric diet). (**C**) Slight accumulation on blood vessel walls (congophilia) in an animal on hypercaloric diet. (**A**–**C**) thioflavin-S staining). Non-transgenic 6-month-old control mice do not show any amyloid deposits. (**D**–**F**) Microglial reaction defined by Iba-1 immunostaining. Semi-panoramic view of the microglial reaction at 6 months of age in the sensory frontoparietal cortex in a control case (**D**), in an APP-PS1 mouse on a normal diet (**E**), and in another transgenic mouse receiving a hypercaloric diet (**F**) Increase in microglial cells and greater expression/accumulation of Iba-1 in (**E**) and, especially, in (**F**) There is an increase in both rounded microglial forms, without extensions, and in forms with abundant and long extensions. (**G**–**I**) In the case of receiving normal food, microglial hyperplasia is greater within or around the amyloid plaques (red circles, (**E**,**G**,**H**)), but in animals receiving a high-calorie diet, microglial hyperplasia is greater in the area of the neuropil free of amyloid deposits. Most plaques are not invaded/surrounded by microglial cells (**I**) (blue circles). In these diabetogenic-induced animals, microgliosis is mainly observed as persistent accumulation of cellular forms with large extensions (**I**) BAR (microns): (**A**,**B**) = 500; (**C**–**F**) = 100; (**G**,**I**) = 50; (**H**) = 25.

**Figure 12:**
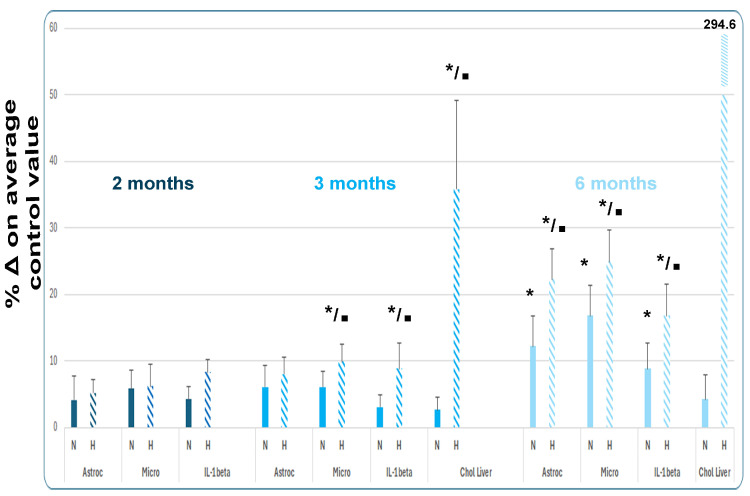
Effect of long-term hypercaloric diet on neuroinflammatory markers in long-term hypercaloric diet/experimental Alzheimer’s model (APP-PS1). Graph showing the percentage increases in neu-roinflammation markers (astrocyte density = Astroc; microglial cell density = Micro; IL-1beta con-centration in cerebral cortex tissue = IL-1beta) in transgenic mice fed a normal (N; blue bars 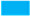) or hypercaloric (H; blue–white bars 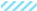) diet versus the mean values given by wild type (non-transgenic) controls. Cholesterol concentrations in the liver (mg/g liver tissue) (Chol liver) are also shown to show damage to peripheral tissues (increasing “metabolic disease” in peripheral organs), which may secondarily increase neuroinflammation. In the groups treated with a hyper-caloric diet, there are statistically significant differences versus the normal control (asterisk) and statistically significant differences versus genetically modified mice fed a normal diet (black square).

**Figure 13:**
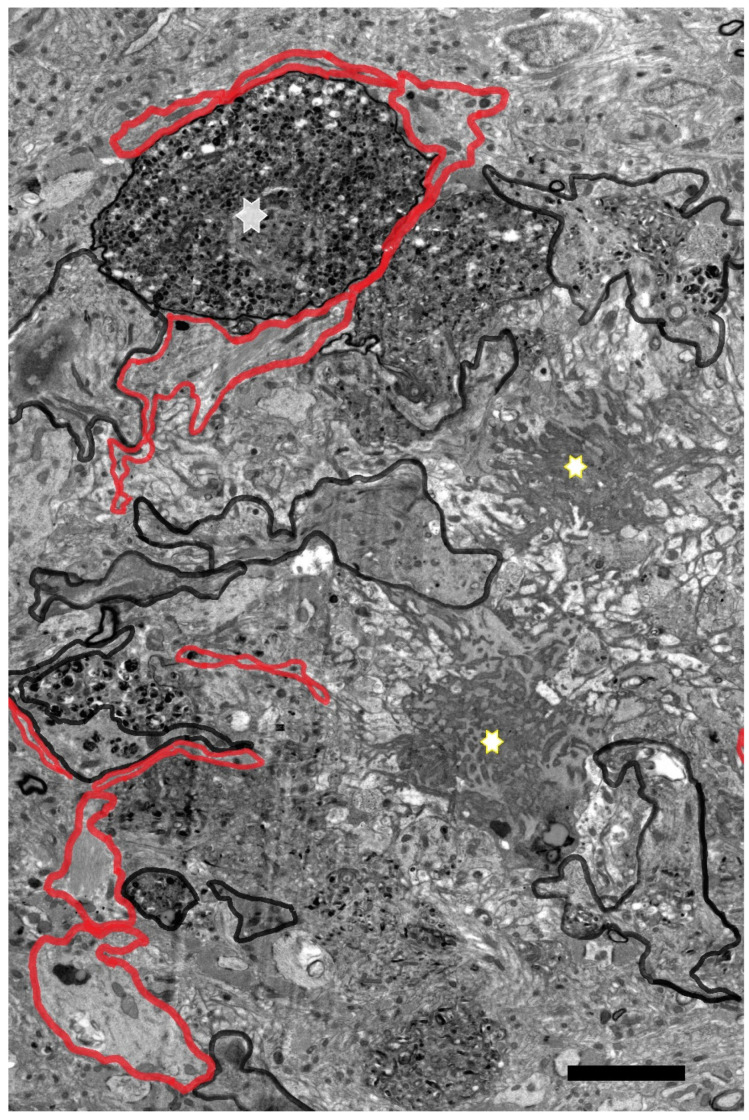
**Electron microscopic image of plaques in long-term hypercaloric diet/experimental Alzheimer’s model** (**APP-PS1**)**.** Two closely related amyloid plaques (turquoise stars) are shown with an electron-dense stellate center with radial extensions of similar appearance and surrounded by small areas of lesser or no electron density. Peripherally, neuronal structures (dendrites, axons, neuronal bodies, and axodendritic complexes with numerous synapses) can be seen with many signs of atrophy and dystrophy, with a large accumulation of autophagosomes and remains of subcellular organoid involution. The image highlights an enormous, myelinated axon (white star) completely filled with subcellular debris. Well-recognizable astrocytic processes have been indicated in red, as well as microglial processes in black (especially from phagocytic microglial elements). There is also free cellular debris in the neuropil. BAR: 70 microns.

**Figure 14:**
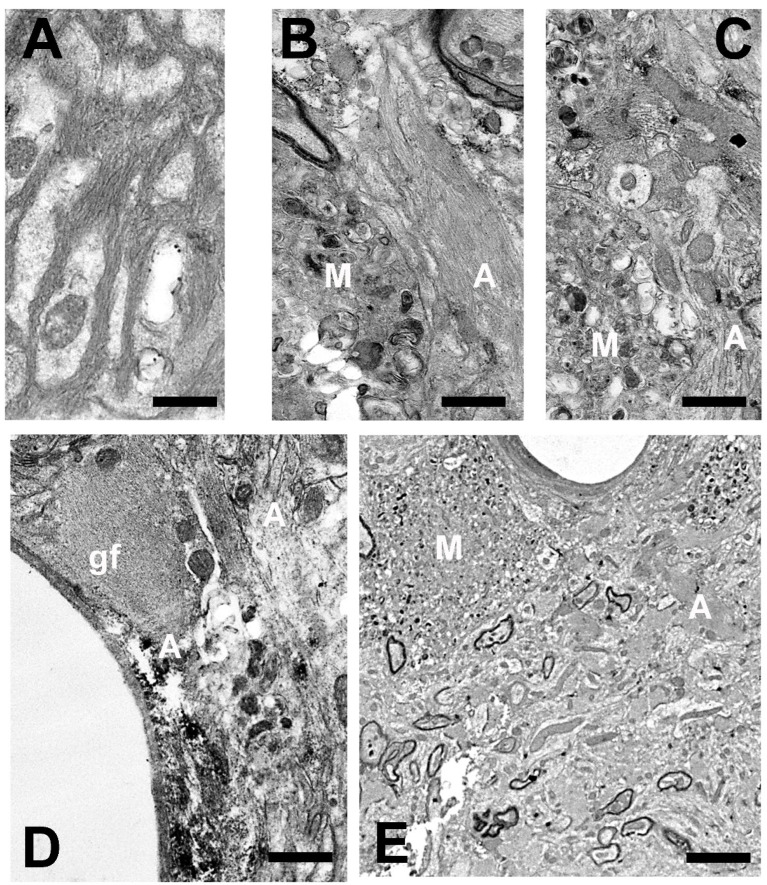
Electron microscopic images of neuroglial processes in long-term hypercaloric diet/experimental Alzheimer’s model (APP-PS1). (**A**) Thin sheets of amyloid fibrillar material, similar to the observed extensions of the core of amyloid plaques, are intermingled in the neuropil of areas peripheral to the amyloid plaques. (**B**) Thick sheets of amyloid fibrillar material, similar to the observed extensions of the core of amyloid plaques, are associated with hypertrophic extensions of microglial cells in the neuropil of areas further away from the amyloid plaques. (**C**) A structure similar to that presented in B but which also includes axo-dendritic formations of normal appearance. (**D**) Hypertrophic but highly degenerative astrocyte with hypertrophic condensations of highly condensed gliofibrils (gf) and abundant lysosomes and residual bodies of distinct types surrounding a dilated blood vessel that presents a thinned endothelium and with signs of cellular involution next to the thinned basement membrane in an area with low density of amyloid plaques. (**E**) Neuropil of an area with low density of amyloid plaques, where a highly dilated blood vessel but of normal structure is observed. Phagocytic microglial cells (M) and hypertrophic astrocytes with abundant gliofibrils (**A**) or varicose processes are observed in the surrounding neuropil. A = astrocyte; M = microglial cell. BAR (microns): (**A**) = 1; (**B**–**D**) = 6; (**E**) = 20.

In this field of research, we have studied (data on the material and methods of this research project are presented at the end of the paper in an Appendix A) the effect of long-term high-caloric diet adherence (47% carbohydrates, 21% fat, and 1.3% cholesterol for 5 months—from 1 to 6 months of age) on brain neuropathology (especially gliopathology) in an experimental Alzheimer’s model, APP-PS1, double-transgenic mouse (APP- Mo/HuAPP695swe/PS1-dE9) [32]. The first gene increases the production of APP, and the second drives metabolism through the amyloid pathway, increasing the levels of the Abeta 1–42 monomer. This peptide led 2o the production of amyloid plaques that begin to appear at 2–3 months of age and grow in size and number in the following months. At 6 months of age, they already present a significant pattern of amyloid accumulation in almost all brain regions but with very different characteristics (large amyloid plaques of various types, smaller granular deposits in the neuropil, and slight accumulation on the walls of certain blood vessels (congophilia) (Figure 11, Figure 12, Figure 13 and Figure 14). Wild-type controls, 6-month-old non-transgenic control mice, do not show any type of amyloid deposits or changes in vessels and perivascular spaces at 3 or 6 months, regardless of the diet received. There were diverse types of plaques in transgenic animals under both diets, similar to what is observed in human Alzheimer’s (although the plaques are not completely comparable between mice and humans), but the number and size were increased in animals with a hypercaloric diet (Figure 13). The amyloid microdeposits on the vessels were similar in both human and other AD model cases, but the intense and persistent congophilia associated along selected vessels is typical of the model (especially under a hypercaloric diet), not to the human beings. Among other neuropathological features, the astroglial and microglial response stands out, the latter much more pronounced than that observed in human patients. The transgenic mice fed the high-fat/caloric diet show, at 2 and 3 months of age, only a slight increase (compared to the average data of control WT animals receiving a normal diet) in astroglial cells (not significant, but very evident in selected mice); at 3 months of age, a significant increase in microglial cells; and at 6 months, a significant increase in both the number and the reactivity of these two types of neuroglial cells (Figure 12). Reactive astroglial cells (GFAP, C-1 complement) appear to be similar (in number, morphology, and location) in the animals that receive the two different types of diets at 2 and 3 months of life, although there are greater numbers of hypertrophic-GAFP hyperreactive astrocytes in the mice that receive a diabetogenic diet for 6 months. Microglial cells increase in number (at 3 and 6 months) either due to an increase in “resident” microglia and/or by invasion of macrophages and transformation into microglial cells (see Section 3.2.1) (Figure 12). Microglial cells react in a differential way according to the diet received. In the case of receiving normal food, microglial hyperplasia and/or cell density are greater within or around the amyloid plaques (Figure 11). We observed that the majority of the plaques (>74%) are closely related (invaded or surrounded) to microglial cells. These cells have variable morphologies: hypertrophic with increased extensions, hypotrophic with decreased extensions, rounded elements with few extensions, and signs of accumulation of substances (phagocytosis). This last morphology is the most prominent. However, in animals receiving a high-caloric diet, microglial hyperplasia is greater in the area that is neuropil-free or little affected by amyloid plaques (>22% compared to mice under a normal diet), and there are many plaques not invaded and/or not surrounded by microglia cells (>44% compared to 26% in a normal diet). In diabetes-induced animals, microgliosis is presented as an increased number of cellular forms with large extensions that are neuropil-free or little affected by amyloid plaques (Figure 11 and Figure 12). These different morphological types of microglial cells share some of their functions (especially those related to the production of pro-neuroinflammatory substances), while they differ in others (rounded shapes may have greater phagocytic functions).

The study of this model, using electron microscopy, has provided a wealth of data to understand the alterations in nervous tissue in AD and T2D-AD. Figure 13 and Figure 14 show alterations in astroglial and microglial cells that support neuroglial dysfunctions.

The results can be interpreted in the sense that the diabetogenic diet modifies the neuroinflammatory response (astroglial and, especially, microglial) in a way that increases the production of pro-inflammatory substances (cytokines) and pro-inflammatory cells (microglia) but reduces the intent (?) of elimination of toxic amyloid products. There are different major subtypes mentioned of microglia associated with the distinct types of plaques, but it is difficult to establish patterns in different regions or layers of the brain cortex or hippocampus. Regional and/or local differences are always observed. It does seem that there are areas, randomly located, with a greater or lesser amount of hypertrophic or phagocytic microglial cells or with diverse types of microglial elements associated with amyloid plaques. This type of neuroglial/neuropathological presentation confirms our descriptions of the variety and diversity of neuroglial response in humans that we have described in several studies [19,20], and that complicates the understanding of the neuropathological course of AD, or T2D, as well as the identification of useful therapeutic targets. For example, in a general sense, hyperglycemia and insulin resistance are well known inducers of microglial changes contributing to AD pathology [94], but it is extraordinarily difficult to fine-tune the therapeutics of microglia polarization to prevent diabetes-associated cerebral alterations [459]. Many studies with recent technologies are necessary to better differentiate neuroglial responses to establish new therapies. This topic will be discussed in Section 5.

Other authors, working with a similar model of AD with a high-fat diet, do not consider that there is astrogliosis or microgliosis at 3 months of age. However, this early gliosis has been observed in other models, indicating the importance of the neuroinflammatory process in the development of neuropathology. The different and/or variable localization of microglial cells in relation to amyloid plaques may indicate the existence of different reactivities (pro-inflammatory and phagocytic) in AD and T2D, although the intense glial reactions coincide when there is comorbidity. It is unquestionable that feeding a high-fat diet accelerates the development of peripheral and central insulin resistance and inflammation and worsens AD-like pathology [439,448]. It should also be noted that six-month-old animals, with 5 months of a diabetogenic diet, show a remarkably high level of hepatic dysfunction/alteration (“fatty liver”, an exaggerated increase in lipid markers), which can be a very important cause of neurodegeneration (Figure 12). The causes and mechanisms of brain deterioration should be studied in more depth.

(B) In the second topic, it has been found that antidiabetic treatments can reduce the pathological alterations in AD models, confirming the consideration of AD as a T3D despite all the reservations that many authors maintain (see Section 2.2). Studies with insulin treatments, as with other antidiabetic drugs, indicate this [57,95,420,460,461,462,463]. Many of these studies have not focused specifically on the behavior of neuroglial cells, but neuroinflammation is always considered one of the main pathogenic mechanisms to regulate/treat the disease. It must be highlighted that the therapeutic results obtained in T2D, AD, and T2D-AD models through diet or lifestyle control (exercise), which are closely related to the control of the reactivity of neuroglia cells, are of great importance in the prevention/treatment of neuropathology [20,464,465]. Gliotherapy will be analyzed in the next section.

We can conclude that the combination of systemic aggression toward the CNS and neuroinflammation induced in the brain itself produces a scenario of mixed AD pathology and cerebrovascular dementia, which, in different regions of the brain tissue, shows more or less pronounced signs of these two pathologies, without many studies having a clear conclusion about the main etiology.

## 4. Neuroglial AD and T2D Therapies

AD, obesity, and T2D have been the subject of a vast number of scientific studies dedicated to their treatments that have led to the publication of hundreds of thousands of papers and review articles and, consequently, to the launch of thousands of clinical trials. However, the therapeutic results in diagnosed patients are far from reaching open expectations with advances in the knowledge of pathological mechanisms and the identification of targets to combat these diseases, and this is despite the advances in pharmacotherapy and cell and gene therapies.

In this review, we have highlighted that glial cells are of capital importance in the pathogenesis of AD, obesity, and T2D, and that, therefore, they can be important targets for the treatment of these diseases. The theory is increasingly becoming established that the treatment of any pathology must be conducted in a multimodal manner, with multidisciplinary approaches that include both preventive and curative interventions [7,20,465]. The multimodal treatment of AD includes the following: (1) non-pharmacological preventive interventions (correcting poor nutritional/diet and lifestyle habits—a sedentary lifestyle, physical exercise, alcohol, and tobacco), etc., such as establishing adequate, and correctly followed, treatments for concurrent pathologies, especially the components of metabolic syndrome). (2) Gliopathological treatments. (3) Neuroprotective/neuroreparative treatments (including the classic “symptomatic” ones, the new cellular or genetic interventions or the new therapies developed in recent years, “medicines modifying the course of the disease” (any effective treatment that improves a predictable pathological course in a patient is not a “medicine modifying the course of the disease”?), based on monoclonal antibodies against amyloid fibers; amyloid deposits; posho-tau; pro-inflammatory molecules; or anti-amyloid-, anti-tau-, or anti-neuroinflammatory “vaccines” [465,466]. We included in Section 3 a large number of preclinical studies carried out by numerous laboratories in the world, which are expected to move to clinical trials in the very near future. In multimodal therapeutical approaches to obesity/T2D, both pharmacological and non-pharmacological interventions must be considered, as well as the use of new cellular and gene therapies [56,57]. In these therapeutic approaches to AD and T2D, actions aimed at normalizing neuroglial (potentially or actually) neurotoxic reactions must be important [465]. Although, at this time, there are no truly effective treatments to counteract the pathogenic effects of neuroinflammation in the CNS (the main deleterious effect of the abnormal reactive cells or the fracas of the neuroprotective/neuroreparative adaptative response), there are important studies (clinical trials and scientific works) that open up possible treatment avenues for AD, obesity, and T2D based on neuroglial interventions. Using the new concept of “treatments modifying the pathogenic course of diseases”, we believe that interventions on the behavior or reactivities of glial cells can change the pathogenic course of the diseases considered in this work. Many works are dedicated to trying to regulate the neuroinflammatory process, acting on astroglial cells, pre-oligodendrocytes, or microglia cells. Very varied approaches are being evaluated, both by acting on receptors or signaling pathways of the neuroglial cells themselves [353,465,467,468,469,470,471] by acting on systemic regulators of these cells (based on the crosstalk of astrocytes and microglia—[122,124]) and using cell-modifying responses or epigenetic modifiers. A substantial number of substances (simple molecules or macromolecules—vaccines and antibodies) that can theoretically change neuroglial responses toward beneficial anti-inflammatory phenotypes in long-term preclinical studies can induce neurotoxic neuroglial responses. The use of granulocyte-stimulating factor, or the increased expression of the macrophage colony-stimulating factor receptor, seemed particularly useful in modifying the response of microglial cells toward phenotypes that increase the phagocytosis of aberrant protein deposits, but other reactive responses were later shown to be neurotoxic [472].

Remarkably interesting therapeutical ways are those dedicated to regulating/normalizing neuroglial functions. Lepiarz-Raba et al., 2023 [473] point out three different metabolic manipulations in enhancing the microglial phagocytosis of Aβ: activation of the PI3K/AKT/mTOR pathways, enhancing mitochondrial OXPHOS, and insulin-restoration effects. The authors suggest that supplementation with lactate, ketone bodies, PUFAs, flavonoids, or NAD+ can enhance microglial phagocytosis with a moderate or transient increase in their inflammatory responses. The regulation of microglial autophagy should be considered both for prevention and treatment in AD and T2D acting on microglial receptors such as TREM2, TLRs, and P2Rs [353,474].

Without intending to be exhaustive in neuroglial therapies, we do want to point out some important results of studies that indicate the great interest of therapeutic interventions that try to normalize neuroglial reactions. We point out below (a) pharmacological studies that improve or correct neuroglial reactions, (b) therapeutic or genetic interventions that attempt to improve neuroglial responses, and (c) non-pharmacological therapeutic interventions that positively modify toxic neuroglial reactions (Table 1).

(a) Neuroglial pharmacological interventions

Table 1 summarizes some types of treatments that are effective in AD, obesity, or T2D and that have some type of action on neuroglial cells in preclinical studies. Moreover, data on authorized clinical studies (ClinicalTrials.gov) are provided.

It is not easy to systematize the drugs or therapeutic interventions that may be useful to prevent neuroglial neurotoxic reactions or normalize neuroglial functions since there is a complex network of possible therapeutic targets that are not yet well defined (specific to a neuroglial type or subtype or reactive phenotype) or that can give rise to harmful secondary phenomena. Most targets have an extraordinarily complex regulation, both intracerebral and systemic, which complicates the desired action of the drug. In the table, generic anti-neuroinflammatory drugs that act on various targets have been considered if, more specifically, they try to normalize the function of specific types of neuroglial cells (or eliminate toxic cells, as in the case of pro-oligodendrocytes). Among the generic regulators of neuroinflammation, we have considered the following in the table: NSAIDS and glucocorticoids (classic anti-inflammatories used in numerous pathological processes); melatonin; antibiotics such as ciprofloxacin, levofloxacin, tetracycline, doxycycline, and minocycline; regulators/inhibitors of the secretion of pro-inflammatory cytokines (such as interleukin IL-2); and blockers of cytokines or their receptors (such as TNF alpha and IL-1 beta) (Table 1). In the table, some medications (or medications in the process of being authorized) that have a greater impact on astrocytes, oligodendrocytes, or microglia cells are shown (Table 1).

Astrocytes have been considered regulators of brain metabolism, the main elements for eliminating some neurotoxic cell-derived products and cells possessing the main potential neuroprotective for brain tissue, etc., but always without forgetting that a neurotoxic response can worsen the pathological process that is to be treated (see Section 3.1 and Section 3.2.1). Consequently, astrocytes are studied as targets for therapeutic action on AD and T2D. Different antidiabetic drugs have been shown to improve brain glucose uptake by acting on astroglial cells. As an example, it can be noted that metformin, the treatment of choice in T2D, increased glucose consumption and lactate release in astrocytes [748]. Likewise, GLP-1 analogs improve markers of inflammation and oxidative stress in normal human astrocytes subjected to various glycemic conditions [749]. Insulin and insulin-sensitizing drugs have therapeutic value in fighting against insulin resistance, which also affects astrocytes [469] (Table 1).

Oligodendrocytes (including pro-oligodendrocytes) have an important and undoubted pathogenic role in both AD and T2D, as mentioned, but they have not received special attention in terms of being important targets for therapeutic actions. The most important studies, later accompanied by clinical trials on dasatinib, have tried to eliminate neurotoxic pro-oligodendrocytes in AD (Table 1).

Microglia cells possess an important collection of therapeutic targets that are waiting to be conveniently explored and exploited to treat many diseases, especially AD, obesity, and T2D. Their ability to conduct a variety of functions helps in the physiological adaptation of neurons and neuronal circuits to control the brain environment. Being the basis of immune defense and eliminating neurotoxic protein products/deposits, they deserve to be the basis of the prevention and treatment of CNS diseases. But in addition, their pathogenic capacities deserve special dedication in the search for therapeutic targets to stop the triggering of neuropathogenic mechanisms (such as those underlying AD and T2D). Transcriptional changes in responses of microglia of murine models of AD and neuroinflammation (465 differentially expressed genes in a study by Shippy et al., 2022) [750] present a high number of molecular functions and pathways of phagocytosis and pro-/anti-neuroinflammatory mechanisms/products useful to develop effective treatments. In this same sense, an observational clinical trial is currently being developed in humans to define important genes and molecules to develop new glial therapies. Table 1 mentions microglial pharmacological interventions: PPAR modulators that reduce microglial activation [751]; TREM2 and TLR inhibitors that decrease the production of microglial cytokines and, at the same time, induce a phenotypic change from the M1 response toward gene expressions typical of the M2 phenotype [528] or phenolic compounds [637,752] (Table 1).

Looking at Table 1, some clear inconsistencies can be seen between the studies, both in the preclinical studies in AD and T2D and between the preclinical studies in each disease and the corresponding clinical trials. Considering the intimate relationship in the pathogenic mechanisms between these two pathologies, there is no great correlation between preclinical studies in both pathologies aimed at finding new medications, nor in clinical trials. Also noteworthy are the enormous differences between the number of clinical trials dedicated to a possible neuroglial drug in AD or T2D and in other more or less related pathologies that have similar pathogenic mechanisms or therapeutic targets. As an example, we can point out that there are 404 clinical trials on ibrutiniv, which decreases the neuroinflammatory response of mesodermal cells (in the case of microglia) but has only been favored by one study in the AD-T2D complex. In the same way, there are many preclinical works that show that the inhibition of TEMP2 and TLR4, conducted by more than 40 substances, favors microglial evolution toward anti-inflammatory M2 phenotypes. There are no clinical trials referring to these pathologies, but there are some referring to others. It can be concluded that the results of the research cannot be assessed by the number of preclinical or clinical works, and only the impact of one of them will make the therapeutic elite of a pathology jump to a substance, even if it comes from another pathogenic field of study.

(b) Cellular or gene therapeutic interventions that act on neuroglial cells

This type of therapy has been developing for a few years but has not yet reached the impressive possibilities theoretically attributed. However, based on the results of solid research work, pluripotent stem cells [753,754] may be the basis of possible therapies in the coming years to “reformat” the aberrant neuroglial cells (Table 1). In these fields of neural therapies, we must not rule out the possibility of inducing “in vivo” the conversion of astrocytes into neurons or oligodendroglia cells to repair damaged neuronal circuits [755,756,757,758]. The astrocyte-derived extracellular vesicles, as well as those of neuronal or microglial origin (normally produced or modified), could be utilized for therapies [759,760,761,762,763]. In this section (Table I), we must highlight the studies (some already in clinical trials in humans) to try to functionally reconstruct the altered cellular organoids in neurons and neuroglial cells. In particular, we must mention the studies and clinical trials aiming to recover mitochondria. There are various therapeutic approaches to achieving this goal: mitochondrial preparations and embryonic cell transplants that enhance mitochondrial biogenesis.

(c) Non-pharmacological therapeutic interventions

In AD, obesity, and T2D, for many decades, scientists and clinicians have been interested in the need to apply non-pharmacological interventions to treat these diseases. The results of clinical trials and published works on the subject showed beneficial results when nutritional guidelines and/or healthy diets and a healthy lifestyle were followed (Table 1), practicing physical exercise proper to the age and constitution of each individual, and avoiding inactivity (a sedentary lifestyle) [764,765,766]. Exercise can be more effective than diet control in preventing amyloid deposition in diabetogenic-treated AD models [767]. Different studies on the subject have shown that these lifestyle rules/protocols normalize astroglial and microglial reactive activities. And this is both in AD and in obesity and T2D, as can be seen in the clinical trials (table).

Cognitive-intervention strategies have proven to have benefits in AD [768], and educational intervention have been extremely useful in the prevention and treatment of obesity and T2D (Table 1).

All of these treatments presented in Table 1 are already being used successfully in many cases, helping to treat the cognitive deficits and neurodegeneration in T2D-AD in an integrated/multimodal manner. Cellular and genetic neuroglial treatments are providing new advances every day. Some scientists consider that neurons could be recovered from neuroglial cells [769].

## 5. Discussion, Conclusions, and Future Research

In the preceding sections (Section 3 and Section 4), we presented the neuropathological features and research findings of the brains in aging; obesity; and T2D, T2D-AD, and AD, both in humans and in experimental models of each pathological group. This review has focused especially on the role of neuroglial cell dysfunctions/responses, which we consider to be of capital importance from the origin to the end of all the pathological processes studied. In a T2D-AD mouse model (APP+PS1 mice on a high-calorie diet) we investigated, we showed that neuroglial dysfunctions (astrocytosis and microgliosis) manifest before the appearance of amyloid neuropathology and that the amyloid pathology is greater than that presented by mice fed a normal, non-high-calorie diet. In the presentation of each clinicopathological set/disease/syndrome, some critical considerations of the referred facts or their interpretations are included. In this section, we want to highlight and discuss, in a more panoramic way, some general or particular aspects, which, in our opinion, are of special importance for the development of this scientific field and for the prevention and treatment of these complex and intricately interconnected pathologies (aging is not, per se, a pathology, but it is a risk factor common to the other pathologies).

The most important conclusion of our review is that neuroglial dysfunctions/alterations are a common feature among the pathologies analyzed. This fact, which we will discuss later, is of great importance both for understanding these pathologies and for being able to prevent and treat them. Working to achieve a better understanding of the complex metabolic-induced brain pathology and prevent the healthy brain from systemic and neuropathological disorders based on neuroglial responses is a useful way to design/plan an efficient way to fight against brain involution.

The topics we consider in this section are (a) the degree of the relationship between T2D and AD and its various neuropathological and cognitive interpretations, (b) the T3D concept, and (c) the relationship between the concept of AD as a cerebral metabolic syndrome and other etiopathogenic conceptions of this neurodegenerative syndrome.

(a) The degree of relationship between T2D and AD and various neuropathological and cognitive interpretations

Following the historical course of the etiological and mechanistic pathogenic relationships of AD and T2D, we want to focus on the publications of the 2005-15 decade. An important review by Nelson et al. [104] presents an overview of clinical epidemiological, biochemical, and neuropathological studies that try to resolve the problem of whether the neuropathology of cases of advanced T2D patients presents a picture completely compatible with AD. This review shows that there have not been many studies related to this topic up to that point but that an earlier polarization existed regarding the consideration of brain alteration as (a) a special case of cerebrovascular disease, (b) a special case of AD, or (c) a cognitive deficit different from these classical pathologies. This review shows that vascular alterations and small vascular hemorrhages/infarcts are very important facts in the T2D brain. However, their analysis of the markers of Alzheimer’s pathology does not show significant differences when comparing AD brains and T2D brains. Neuroglial cell analysis has not been considered in these studies. In several studies, it is pointed out that the substantial difference between T2D and AD lies in vascular alteration, which many authors point out as the most relevant fact. It is considered that there are three important aspects in the brains of T2D patients who have cognitive deficits (or AD): disorders related to glucose use, amyloidosis, and cerebrovascular alterations. If we take into account two important clinical pathological facts, such as the verification that a high number of patients with MCI (the previous step to the development of AD) present insulin resistance although without marked peripheral symptoms of inflammation, as well as that many individualized anatomopathological analyses in cases of AD present a varied range of associated neuropathological changes (small vessel damage, synucleinopathy, amyloidosis, etc.) to the markers and signs of insulin resistance, we can consider that patients suffer from a form of T2D-AD that manifests itself in diverse ways depending on the life course of each individual. In most cases, the diagnosis of AD comes from the existence of clinical characteristics compatible with AD conditions, but histological studies show a wide presence of lesions compatible with different diagnoses: (cerebro-)vascular dementia, small vessel damage, or cerebral amyloidosis. In other cases, clinical AD is not evident, but AD markers are significant. Many authors conclude that the majority of T2D-AD brains present very intense vascular lesions and a low grade of typical AD lesions. (Do patients with mixed T2D-AD pathology have an earlier death than AD patients without T2D- or pro-AT2D-induced alterations?) The clinical diagnosis of AD does not seem to be mandatory for the classification of case studies to investigate brain neurodegeneration.

In recent years, many subsequent studies, based on research carried out on human brains and, especially, on appropriate experimental models (T2D and AD), highlighted that brain alterations in patients with T2D and/or AD show very similar characteristics when analyzing neuroglial alterations and the underlying pathogenic mechanisms.

**Figure 15:**
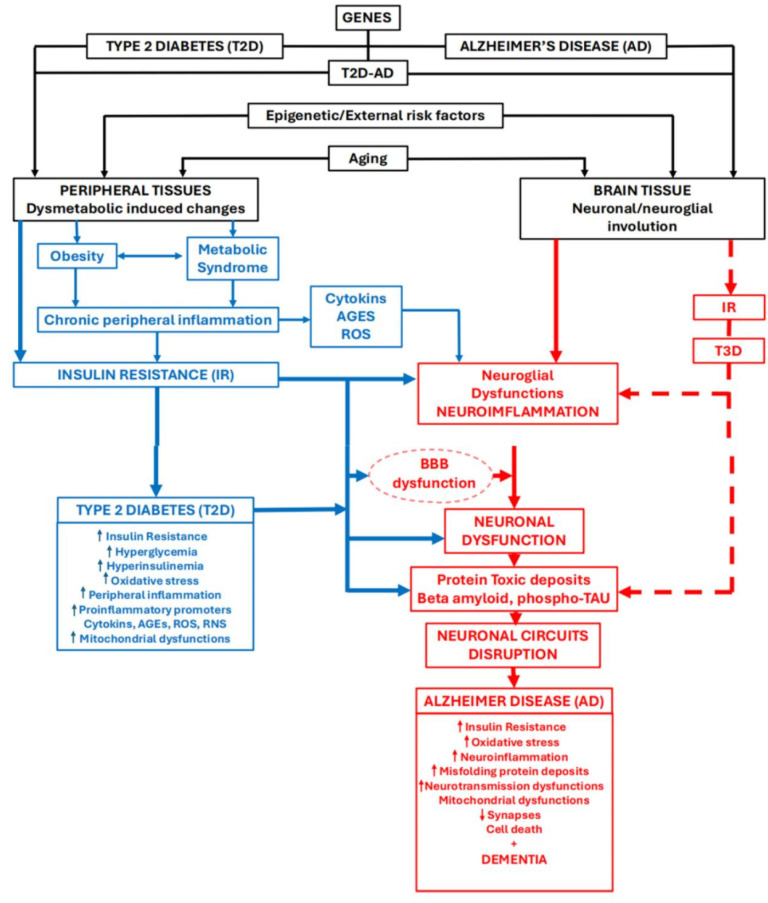
**Diagram showing the relationship between T2D and AD**. T2D and AD have close pathogenic connections. There are genes involved in the development of each of these pathologies and others common to both. External risk and epigenetic factors are similar in both cases, and aging is an inducer of both pathologies. T2D develops mainly in peripheral tissues when metabolic changes occur (left side, in blue). Obesity and/or the various forms of metabolic syndrome condition a state of chronic peripheral inflammation that, through cytokines, AGEs, and ROS, induce the first dysfunctional changes in the neuroglial cells of the brain that initiate neuroinflammation. In any case, the main pathogenic mechanism of peripheral dysmetabolism is the development of insulin resistance that leads to T2D with all the components indicated in the diagram. AD (right side, in red) develops mainly after a process of neuroglial dysfunction that induces neuronal dysfunction, with the appearance of beta-amyloid and phospho-tau protein deposits. In AD, the same components that are indicated in T2D appear in the brain, in addition to specific synaptic and neurotransmitter alterations. Insulin resistance and the development of T2D induce significant AD-inducing changes in the brain, especially neuroglial dysfunction, with the subsequent neuroinflammatory process that is key to neurodegeneration, as well as neuronal involution and promotion of the formation of beta-amyloid and phospho-tau. The possibility that AD may develop as a type of cerebral diabetes (T3D) induced by insulin resistance developed specifically in the brain is also considered (see text).

In Figure 15, we compile a diagram of the key pathogenic mechanisms and relationships in the T2D (and related dysmetabolic processes) and AD interconnection. Interconnection can occur at different stages of the individual’s life (whether or not he or she suffers from obesity, T2D, or metabolic syndrome; whether a process of brain neurodegeneration has already begun). T2D can be the initiator of brain neurodegeneration toward AD, a collaborator in the pathogenic course or an accelerator of the process in one of its phases. It has been found that many patients with MCI have metabolic alterations and insulin resistance. On the other hand, studies carried out with animal models of T2D, AD, and T2D-AD have shown, with greater precision, the pathogenic mechanisms and their key points to cause pathological changes and/or show possible therapeutic targets. It is also worth noting that in all these recent studies (with greater relevance in research on models), the capital importance of glial dysfunctions and neuroinflammation (from the origin to the final phases) in the morphofunctional changes that underlie cognitive deficit/AD/dementia has been demonstrated. Astrogliosis and microgliosis are present in the above-mentioned T2D-AD model that we studied before the significant manifestation of beta-amyloid deposits and congophilia (at two months of life and one month of a high-calorie diet), as well as a greater manifestation of these deposits at six months of life (and five months of a high-calorie diet) (see Section 4).

To understand the close relationship between T2D and AD, genetic research is very relevant. A large number of alterations in the expression of genes using transcriptomics/gene expression modification analysis in the temporal cortex of T2D patients (with a very limited degree of clinical and neurological cognitive alteration suggesting AD) have been found not only in neurons but in astrocytes and endothelial cells (which are considered to support the glial and vascular involvement of T2D in the involution/degeneration of the brain) [105]. Gene expression changes are detected in neurons, including alterations in insulin and other signaling pathways, the cell cycle, cellular senescence, inflammatory mediators, and components of the mitochondrial respiratory electron transport chain. However, more genes (2202 and 1227 genes) were significantly different expressed in astrocytes and endothelial cells, respectively. Changes in cortical impaired insulin signaling were shared by neurovascular unit cells with, additionally, apoptotic pathway changes in astrocytes and dysregulation of advanced glycation end-product signaling in endothelial cells. In general, neuropathological studies in non-demented humans indicate a lower neurodegenerative reactivity compared to the studies carried out on the brains of AD patients (with amyloid plaques and phospho-tau accumulations). In certain studies [32], a lower presence of microglia in the vicinity of amyloid plaques, which can be interpreted as a decrease in the destruction/phagocytosis capacity of these potentially neurotoxic accumulations, has been described (similar to our results in the T2D-AD model).

All of these results described and analyzed in various fields (epidemiological, clinical, and neuropathological) pose a problem of interpretation based on varied factors. We want to point out three of them:

First, it must be considered that AD is a syndrome of diverse and unknown etiology. It is necessary to review the various genes involved in the genesis and development of not only familial AD and sporadic AD but also the involvement of other different risk factors (diet and lifestyle) that induce epigenetic changes [770]. It is necessary to perform more transcriptional studies to know the exact phenotypes of the neuroglial cells in each brain region in every phase of the course of the disease for a “personalized” therapy.

Second, we must also consider the differences that may exist (mentioned above) in the diverse characteristics of human brains studied under the general classification of AD-T2D: the duration and characteristics of the underlying metabolic disease (pre-diabetes, obesity, systemic lesions/dysfunctions) and the duration and characteristics of AD neurodegeneration—prodromal AD and MCI. Several authors have pointed out that it is possible that the coincidence of T2D and cognitive decline/pre-AD can reduce the life expectancy of individuals who suffer from it and, in consequence, fewer neuropathological alterations can be observed in T2D-AD brains than in AD brains.

Third, it should be noted that T2D patients have generally received adequate and effective treatments (something that does not currently exist in AD); hence, their latent disease, T2D, could be damaging the brain for many years, causing neurodegenerative changes in the brain and other organs that lead to a more premature death than that induced by the development of AD as the only important pathology. Studies on T2D models on the course of the disease show highly intense neuroglial responses, but the effectiveness of various treatments displays a decrease in the neuropathology at death. This becomes more evident in AD and AD-T2D models. In these models, it is worth highlighting the almost constant confirmation of the increase in astrogliosis and microgliosis, more striking in AD models in which T2D is induced. Microgliosis in AD-T2D models induced by a high-fat/glycose diet is evidenced by an increase in the number of microglia cells and cytokine levels in the cerebral cortex and hippocampus long before they appear the characteristic markers of AD (amyloid plaques and neurofibrillary tangles). This scenario persists in the phase with a significant presentation of AD pathology markers, although the close relationship of neuroglial cells with amyloid plaques typical of human AD pathology is not always seen in these models.

(b) The T3D concept of AD

As mentioned, the concept that AD was a metabolic disease arose from the initial observation that, in AD, there is a glucose dysmetabolism (later extended to lipid metabolism) that is induced by peripheral inflammatory mechanisms and, as a key element, the development of insulin resistance (Figure 15). This expands or justifies, according to the interpretations of different authors, the initial theory of the “pathogenic amyloid cascade” that leads to AD. Insulin resistance and associated alterations (hyperinsulinemia and hyperglycemia) in the brain are observed in some cases of human AD and in some AD models (although not well studied) without there being a clear manifestation of peripheral inflammation or metabolic disease. Considering insulin resistance and related alterations to be very important in AD neurodegeneration, the name type 3 diabetes was proposed for this situation. It could be compared to one of the possible components of the metabolic syndrome that develops preferentially in the brain rather than in a peripheral organ. However, this concept should be revised since there are indications that an isolated IR does not occur in the brain but that this pathogenic mechanism is correlated with other changes that occur during aging. On the one hand, it has been proven that there is a real alteration in glucose metabolism during aging that, without being clinically diagnosed or treated, induces a “pre-diabetic” alteration, as it has been called by various authors, and that can be detected (although with difficulty) in the brain and peripheral organs. Moreover, there is a marked increase in the number of senescent cells (neuronal, astroglial, oligodendroglial, microglial, and endothelial–cerebral) in the brains of T2D, AD, and T2D patients [771,772,773]. These senescent cells, induced by cellular stress and which have an important implication in neuroglial and neuronal dysfunctional alterations, may have been activated in their senescence pathway by glucose dysmetabolism and its consequences [773]. In an AD model mouse, removing senescent cells (genetically or pharmacologically) ameliorates beta-amyloid peptide and tau-protein-induced neuropathologies.

(c) The relationship between the concept of AD as a cerebral metabolic syndrome and other etiopathogenic conceptions of this neurodegenerative syndrome.

“Alzheimer’s disease” is actually a complex multifactorial syndrome defined primarily by an “amyloid pathogenic cascade”, later extended to other cascades of misfolding proteins and extended/reinforced with metabolic disorders, as explained in previous sections. However, this theory can be considered contrary to other theories on AD.

We consider that although a unitary theory cannot yet be proposed (and may never be achieved, as it is a multifactorial syndrome), many etiopathogenic theories and key mechanisms in the development of AD are not far from the conception of AD as a metabolic syndrome. First of all, one must consider the difficulty that exists in differentiating “causes” from “effects” in the different theories stated. The same mechanisms can be “placed” in different preferential orders in different proposals of pathogenic courses.

On the differences with other theories on AD, we would like to comment on the following:

The theories on oxidative stress, amyloid alterations (abnormal production, accumulation, or removal), tau proteinopathy, mitochondrial dysfunctions, cerebral vascular and BBB dysfunctions, and inflammation/neuroinflammation are intimately involved in the consideration of AD as a “neurometabolic syndrome”.

The theories on neurotransmitter dysfunctions in AD are not sufficiently studied specifically in their dysmetabolic relationships, but there are works that indicate that neurotransmitter systems are highly regulated by metabolic changes. The cholinergic system is regulated by neuronal metabolism as well as the glutamatergic system and dopaminergic system [774,775,776,777].

- The senescence of all brain cells, neurons, neuroglial cells, and endothelial cells has already been commented on as having relationships with metabolic changes [179,771,772,773].
- The theory of mitochondrial dysfunction is a common link between T2D and AD [778].
- Genetic theories of AD do not conflict with the conception of the mechanistic connection of T2D and AD: many pathogenic mechanisms are reinforced, such as those related to genes affecting microglial cells or mitochondria, also common to T2D and AD. Studies on gene expression in brain cells (neuronal, neuroglial, and endothelial) of AD, T2D, and T2D-AD patients that have been carried out in recent years show that many of the overexpressed or down-expressed genes are related to insulin resistance and changes in insulin signaling pathways [105,774].

Epigenetic theories. A close relationship between T2D and AD has been observed for years and is the basis for a strong preventive therapy [770].

We can conclude that neuroglial alterations (especially astroglia and microglia, the basis of the neuroinflammatory process), are key promoters of brain neurodegeneration via peripheral inflammation inducers and neuropathological-induced aberrant/neurotoxic responses related to insulin resistance in T2D-AD. Even in AD brains with apparently normal peripheral metabolism, there may be small metabolic dysfunctions that directly induce glucose dysmetabolism or insulin resistance (known asT3D) or indirectly through an increase in senescent neuroglial cells (astroglial, oligodendroglial—including pro-oligodendrocytes—and microglial cells) that will lead to AD neurodegeneration. There are several differences between each of the studied pathologies (cerebrovascular component and cognitive decline), but the main deleterious effect on neurons is similar and highly neurodegenerative on the provoking neuronal circuits.

Neuroglial dysfunctions must be studied in depth both to understand the characteristics and circumstances of each pathological process and to obtain effective results in therapies. The double function of the responses of neuroglial cells, both in a “neuroprotective” and “neurotoxic” aspect (without forgetting that the elimination of toxic cells is beneficial for the health of the tissue), requires a clear differentiation of which changes/responses must be encouraged and which must be prevented or counteracted [19,125,729]. The need for further research in two very different directions is evident for a better understanding of these pathologies and for efficient prevention/treatment of these disorders: (a) the characterization of the phenotypic changes of astrocytes and microglial cells in each region of the brain and in each phase of development of each isolated and associated pathology (single-cell studies are mandatory); and, (b) the study of new therapeutic avenues to normalize the function of neuroglial cells (preventing neurotoxic responses and/or reversing them) in these pathologies, along with the phenotypic characteristics in each moment of the diseases course and affected brain region of the mixed neurodegenerative process.

## Figures and Tables

**Table 1:** Treatments and clinical studies directly or indirectly related to neuroglial cells in obesity, type 2 Diabetes (T2D), Alzheimer’s disease (AD), and mild cognitive impairment (MCI). The data in the table have been collected jointly and comparatively between T2D and AD. Pharmacological, cellular, and genetic treatments, as well as non-pharmacological interventions, are reviewed, both in preclinical studies and developed in controlled clinical trials (in this case, the NIH ClinicalTrials.gov website was used—consulted in June–August 2024). Treatment data are accompanied by illustrative bibliographic references on mechanisms of action, preclinical studies, and reviews of clinical studies. The numbers on clinical studies are not necessarily those obtained directly from the ClinicalTrials.gov database, since only studies directly or indirectly related to the CNS and neuroglial cells and/or neuroinflammation are referenced.

DRUG/INTERVENTIONS			COMENTS/OBSERVATIONS	OBESITY/T2D		AD/MCI	
				Ref (PRE-CL + Rew)	CLIN-TRIALs	Ref (PRE-CL + Rew)	CLIN-TRIALs
**GENERIC ANTI-INFLAMMATORY TREATMENTS**			Modulators of pro-inflammatory cytokins secreted by diverse types of neuroglial cells or neurons				
	**NSAIDS and Anti-COX2**		Potent anti-inflammatory drugs in peripheral tissues with action on neuroinflammatory processes. Cooperative drugs in multimodal AD therapies	[475,476,477,478]		[479,480,481,482,483,484,485]	
		Indomethacin	Also targets many proteins in brain	[486]		[487,488,489,490,491]	1
		Diclofenac	Special action on microglia	[492,493]	(Obesity 2)	[494]	
		Celecoxib	Potent anti-COX2	[495,496,497]	2 (Obesity 5)	[498,499,500,501]	4
	**Melatonin**			[502,503,504,505]	6 (Obesity 14)	[506,507,508,509]	7
	**Minocyclin**		Antibiotic (tetracyclin) Anti-inflammatory	[510,511]		[512,513,514,515]	1
	**Interleukine-2**		Brain anti-inflammatory and regulatory of T cells expansion and activation (Tregs). IL-2 induces astrocytic activation and recruitment around amyloid plaques, decreasing amyloid plaque load				
		IL-2 molecule		[516,517,518]		[516,519,520]	3
		AVV- Il-2 gene vector				[520]	
	**Interleukine-1 antagonists**		Interleukin 1beta is the most potent pro-inflammatory cytokine (along with TNF, studied below along with treatments against activated microglia)				
		interleukin-1 receptor antagonist (IL-1RA)	IL-1RA is a human protein that non-productively binds interleukin-1 receptors, preventing IL-1 responses	[521]	8 (Obesity 3)	[521,522,523]	
		IL1-beta vaccine			1		
		IL-1β antibody/canakinumab	It presents problems in the passage of BBB. It is highly effective in reducing TNF-1beta of peripheral origin. There are 111 clinical trials on peripheral inflammation/syndroms	[524,525,526]	5	[524,525,526]	3
	**Cholesterol-lowering drugs**	Simvastatin, Lovastatin	Cholesterol-lowering drugs reduce intracellular and extracellular levels of Aβ42 and Aβ40 peptides and neuroinflammation Well-studied in the brain in AD, not in T2D (where there are instead many clinical trials). It is part of the antidiabetic polypill	[527]	2	[528,529,530,531]	1
	**Polyphenolic compounds**		Polyphenols are natural substances that have several beneficial effects acting on neuroglial cells (astrocytes and microglia): anti-inflammatory, antioxidant, anti-NO				
		Polyphenols/Isoflavones (in general)		[532]	31 (Obesity 65)	[533,534,535,536,537,538]	6 (MCI 1)
		Resveratrol		[539,540,541,542]	16 (Obesity 20)	[543,544,545,546,547,548]	6 (MCI 4)
		Epigallocatechin-3-Gallate		[549,550]	4 ((Obesity 8)	[549,550]	3 (MCI -)
		Epicatechin, catechin		[551,552,553,554]	3 (Obesity 6)	[555,556]	1 (MCI -)
		Quercetin		[554,557,558]	2 (Obesity 4)	[557,559,560,561,562]	6 (MCI 4)
		Curcumin		[553,563,564,565,566,567,568]	10 (Obesity 8)	[569,570,571,572,573]	7 (MCI 7)
		Ginkgo Biloba				[574,575]	
		Ferulic acid				[576]	
		Taxifolin		[577]		[578,579,580]	
	**Vitamins and pro-vitamins**		Vitamins and provitamins have antioxidant and antineuroinflammatory effects on the brain. There are many clinical studies on obesity and T2D since these substances play an important role in the genesis and development of the disease	[581]	200 (Obesity 313)	[582,583,584,585,586,587,588,589,590]	37 (MCI 27)
	**Antidiabetic Drugs**		Hyperglycemia, hyperinsulinemia, and insulin resistance are phenomena shared by T2D and AD (see text). These pathological alterations affect neuroglial cells (mainly astrocytes and microglia cells). Antidiabetic treatments reduce neuroinflammation in AD. Clinical trials are shown here			[57,460,461,462,463,591,592,593,594,595,596]	
		Semaglutide					4
		Liraglutide					2
		Pioglitazone					3
		Rosiglitazone					15
		Insulin					36
		Metformin					4
	**LIFESTYLE INTERVENTIONS**						
	**Diet**		Diverse types of diets (Mediterranean, ketogenic, etc.). Diet is a main regulator of risk factors for T2D and AD diseases, acting on neuroinflammation and metabolic processes	[439,597,598,599,600,601,602,603,604,605,606,607,608]	41 (Obesity 3181)	[567,609,610,611,612,613,614,615,616,617,618,619,620,621,622,623,624]	105 (MCI 77)
	**exercise**		Programmed/regulated. Main component of healthy lifestyle. Exercise regulates neuroinflammatory processes	[625,626]	49 (Obesity 3036)	[627,628,629,630,631,632,633,634,635,636,637]	49 (MCI 273)
	**CELL AND GENIC TREATMENTS**						
	**Stem cell therapy**						
		Astrocyte transplants				[638]	
		Mesenchymal cells of different origins (adipose tissue and umbilical cord)		[639,640,641,642,643,644]	3 (Obesity 2)	[645,646]	27
		Exosomes of stem cells of different origins, including laboratory-manufactured liposomes		[647,648]		[616,649,650,651,652,653,654]	
		Mitochondrial transplants				[655,656,657,658,659]	
	**GENE THERAPY**		Gene vectorization (neurotrophins, APOE				
		AAV-BDNF					1 (MCI 1)
		AAV-NGF					4 (MCI 1)
		AAV-APOE2					2
		AAV-telomerase					1
	**MICROGLIA** **REPROGRAMMING**		Induction of the change from M1 phenotype to M2 phenotype in microglia cells using different methods (drugs, diets, nutraceuticals, and interleukin-3)	[200,660,661]		[662,663,664,665]	
**ASTROGLIA TREATMENTS**							
	**Glucocorticoids**		Antioxidants and anti-inflammatories. Used with caution in T2D due to possible adverse effects				
		dexametasone, prednisone, etc.	Synthetic molecules similar to naturally occurring steroid hormones. Anti-inflammatory, neuroprotectors, inducers of neroplasticity. Protection against AD stress, acting on astrocytes	[666,667]	1 (Obesity 23)	[668,669,670,671,672,673,674,675,676,677,678]	6 (MCI 4)
**OLIGODENDROGLIA TREATMENTS**							
	**Quercetin + Desatinib (senolytic cocktail)**		Decreases the number of neurotoxic/senile pro-oligodendrocytes	[679]	1	[240,680,681]	7
**MICROGLIA TREATMENTS**							
	**Neuroinflammatory microglial modifiers**		Induce pro-neroinflammatory phenotypes to change to anti-neuroinflammatory/phagocytic phenotypes				
		Candesartan	Angiotensin II type 1 receptor blocker. Induces a more anti-inflammatory microglia phenotipe and also reduces NO and TGF- β1 levels	[682,683,684]	6	[685,686,687]	5
		simufilam	Binds to Filamin A, scaffolding protein, and regulator of the actin cytoskeleton. Induces changes from a pro-inflammatory to a phagocytic microglial state: improves mitochondrial function. Filamin has been claimed to stabilize the high-affinity interaction of soluble Aβ42 and the α7 nicotinic acetylcholine receptor (α7nAChR), which has been reported to trigger tau phosphorylation and synaptic dysfunction in some experimental systems			[688,689,690]	7
	**PPAR modulation**		Peroxisome proliferator-activated receptors (PPAR) down-regulate the expression of genes related to microglial activation				
		GF1803	Pan alfa/delta/gamma-PPAR agonist				1
		Pioglitazone	gamma-PPAR agonist	[691,692,693,694,695]	22	[696,697,698,699,700]	7
	**TNFalpha modifiers**		TNF alpha pro-inflammatory cytokin inhibitors				
		Etanercept, Adalimumab	Monoclonal antibodies. TNF alpha receptor blockers	[701]		[702,703,704]	5
		Pegipanermin/CXPro 1595	TNF variant devoid of TNF receptor-binding activity. Blocks soluble TNFalpha-forming heterotrimers			[700]	4
	**TREM2 and TLR 1-4**		Triggering Receptor Expressed on Myeloid cells-2 (TREMP-2) and Toll-like receptors (TLR 1-4) modulation, promoting phagocitic microglial responses and anti-inflammation	[705,706]		[707,708,709,710,711,712,713,714,715,716,717]	
		(ciprofloxacin/levofloxacin/rifampicin)	Antibiotics promoting microglial anti-inflammatory response			[718,719,720]	3
		mesoindigo	Inhibits TLR and inflammasome complex			[721]	
		oxymatrine	Quionolizidine alkaloid. Active on other signaling patways			[722,723,724,725]	
		baicalin	Flavone anti-inflammatory. Also promotes neural proliferation	[726,727]		[728,729,730,731]	
		ibrutrinib	Inhibits Tyrosine Kinases	[732,733]		[729,730,734]	
	**Signaling pathways modifiers**						
	**MAPK signaling pathways**		Diverse MAPK signaling pathway activation may lead to the production of pro-inflammatory cytokines, which promote neuroinflammation				
		Dexmedetomidine	MAPK/ERK1/2 inhibitor. alfa-2 adrenergic receptor agonist. Microglia regulator. Anti-inflammatory. (Clinical trials on neuroinflammation related to surgery)			[734,735,736,737,738,739,740]	14
		VX-745 (Neflamapimod)	p38 MAPK-α inhibitor			[741,742,743]	2
		Baricitinib	MAP3K 12, Jak inhibitor				2
	**NF-κB**		NF-kappaB activation may lead to the production of pro-inflammatory cytokines, which promote neuroinflammation				
		Atomoxetine	Noradrenaline reuptake inhibitor and reducer of activation of NF-kappaB (also acting on astrocytes)			[744]	1
		Oxyimperatorin	Natural product. Suppresses NF-κB p65 signaling			[745]	
		Oxysophoridine	Bioactive alkaloid			[746]	
		Parthenolide	Sesquiterpene lactone acting on diverse sgnaling pathways			[747]	

## Data Availability

The data presented in this review were obtained from the data records and graphic material of our own research, as well as from relevant publications on this topic (obtained through https://pubmed.ncbi.nlm.nih.gov/) and from clinical studies (https://Clinicaltrials.gov) (both accessed from January 2023 to September 2024).

## Appendix A

This table presents a summary of the methodology used in the study of the T2D-AD model presented in Section 3.2.5, neuroglia in human AD-T2D and models.

**NEUROPATHOLOGICAL AND NEUROGLIAL CHANGES IN A T2D-AD MOUSE MODEL (APP+PS1 MICE ON A HIGH-CALORIE DIET)** **Material and Methods (abstract)** ***Animals*** Control mice (wild type—WT): C57BL/6 (males). Transgenic mice: APPswe/PS1dE9 (APP/PS1) transgenic mice. ***Diets tested*:** (a) standard control diet in the animal facility (cd); (b) high-calorie diet: 47% carbohydrates, 21% fat, 1.3% cholesterol (high-fat/hipercaloric diet) (hcd). ***Trial groups (4) and number of animals in each group*:** 1- Control WT animals with a standard diet (12). 2- Control WT animals with a hypercaloric diet (12). 3- Transgenic APP/PS1 animals with a standard diet (12). 4- Transgenic APP/PS1 animals with a hypercaloric diet (12). ***Duration of the trial***: 5 months (from 1 to 6 months of age). The diets were used from the first month of life. The samples were obtained at 2, 3, and 6 months of life of the animals. ***Obtaining and processing of brains and other tissue samples****:* At 2, 3, and 6 months of treatment, twelve animals were randomly selected from each group. Of these animals from each group, six were decapitated, and the brain (from which the frontoparietal cortex and hippocampus were isolated) and the liver were removed. Homogenates were obtained and stored at −20 °C. The rest of the animals were perfused with 4% p-formaldehyde after anesthesia. The fixed brains were sectioned coronally, obtaining serial sections for histological and immunohistochemical staining, as well as thioflavin-S staining. Some small pieces of these sections were impregnated with osmium tetroxide and processed in Araldite to be observed under an electron microscope. Liver samples were processed for optical and electron microscopy studies. ***Histopathological studies and quantitation****:* Various antibodies were used to characterize neurons, astroglial cells (GFAP, C-1 complement), microglial cells (IBA-1, CD 68, lectins), APP, and phospho-tau. Using the Nikon microscope image analysis systems, the number of astroglia and microglia cells, as well as amyloid deposits in selected areas (6 areas per slice × 10 slices) in both the frontoparietal cortex and the hippocampus (CA-1 and CA3), were quantified. ***Statistical study***: The data were obtained from each individual in each group, as well as the mean +/− SEM for each group. For data presentation, the percentage increases in each group over the mean value of group 1 (control animals on a standard diet) were used, considered 100%. Differences between groups were considered significant at *p* < 0.05.
